# Equatorial restriction of the photoinduced Jahn–Teller switch in Mn(iii)-cyclam complexes†

**DOI:** 10.1039/d3sc01506h

**Published:** 2023-05-26

**Authors:** Ryan Phelps, Alvaro Etcheverry-Berrios, Euan K. Brechin, J. Olof Johansson

**Affiliations:** a EaStCHEM School of Chemistry, University of Edinburgh David Brewster Road EH9 3FJ Edinburgh UK olof.johansson@ed.ac.uk

## Abstract

Ultrafast transient absorption spectra were recorded for solutions of [Mn^III^(cyclam)(H_2_O)(OTf)][OTf]_2_ (cyclam = 1,4,8,11-tetraazacyclotetradecane and OTf = trifluoromethanesulfonate) in water to explore the possibility to restrict the equatorial expansion following photoexcitation of the d_*xy*_ ← d_*z*^2^_ electronic transition, often resulting in a switch from axial to equatorial Jahn–Teller distortion in Mn^III^ complexes. Strong oscillations were observed in the excited state absorption signal and were attributed to an excited state wavepacket. The structural rigidity of the cyclam ligand causes a complex reaction coordinate with frequencies of 333, 368, 454 and 517 cm^−1^, and a significantly shorter compressed-state lifetime compared to other Mn^III^ complexes with less restricted equatorial ligands. Complementary density functional theory quantum chemistry calculations indicate a switch from an axially elongated to a compressed structure in the first excited quintet state Q_1_, which is accompanied by a modulation of the axial tilt angle. Computed harmonic frequencies for the axial stretching mode (∼379 cm^−1^) and the equatorial expansions (∼410 and 503 cm^−1^) of the Q_1_ state agree well with the observed coherences and indicate that the axial bond length contraction is significantly larger than the equatorial expansion, which implies a successful restriction of the wavepacket motion. The weak oscillation observed around 517 cm^−1^ is assigned to a see-saw motion of the axial tilt (predicted ∼610 cm^−1^). The results provide insights into the structural perturbations to the molecular evolution along excited state potential energy surfaces of Mn^III^ octahedral complexes and can be used to guide the synthesis of optically controlled Mn^III^-based single-molecule magnets.

## Abstract 2

Ultrafast transient absorption spectroscopy was used to study the Jahn-Teller switch for [Mn^III^(cyclam)(H_2_O)_2_]^3+^ following the photoexcitation of the d*_xy_* ← d_*z*^2^_ electronic transition.

## Graphical abstract

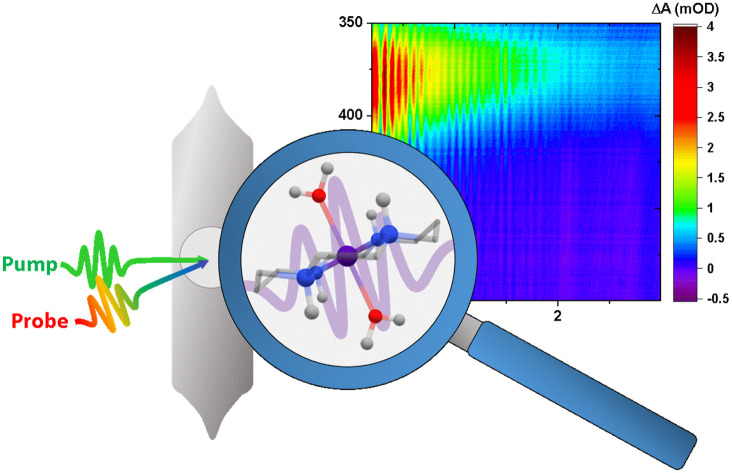

## 1. Introduction

Single-molecule magnets (SMMs) hold great promise for future applications in ultra-dense data storage devices,^1^ owing to their small size and well-defined magnetic properties. Mn^iii^ complexes have often been used as building-blocks for SMMs because they can possess uniaxial magnetic anisotropy, originating from the Jahn–Teller (JT) distortion, which provides a method to store digital information encoded in the direction of the magnetisation (either “up” or “down”).^2–10^ To increase the speed of future data storage devices, new ways of overwriting old data by switching the magnetisation direction is needed. One approach is to use femtosecond laser pulses to change the magnetic anisotropy in the excited state, which in some solids can lead to a full magnetisation reversal *via* either electronic^11^ or phonon^12,13^ excitation. This avenue remains unexplored in SMMs but could enable ultra-dense memories operating on unparalleled timescales. We have previously demonstrated the possibility to use ultrashort laser pulses to influence the magnetic anisotropy, enabled by the JT distortion and the spin–orbit coupling, in Mn^iii^ SMMs.^14^ However, several questions remain open due to the complexity of excited-state properties of SMMs. In this work, we have used a simple Mn^iii^-monomer as a prototype system to provide insights into the coupling between nuclear and electronic degrees of freedom in the excited states of Mn^iii^ complexes. This has allowed us to learn how to control the JT distortion in the excited state, which is a prerequisite for being able to optically switch the magnetisation direction in SMMs.

The photoexcitation of molecules to displacive potential energy surfaces can result in coherent nuclear motion out of the Franck–Condon region.^15^ These vibrational coherences can be observed using transient absorption spectroscopy (TAS), displaying oscillations superimposed onto the kinetic traces of the transient absorption signals. Fourier analysis of the oscillations provides insights into the coupling between nuclear and electronic degrees of freedom, revealing the reaction coordinates involved in photochemical transformations. For example, the reaction coordinates for intersystem crossing (ISC) have been studied for a range of Fe^ii^,^16,17^ Ru^ii^ ^18^ and Cr^iii^ ^19^ complexes. Furthermore, the contraction of the Au–Au bond in photo-excited [Au(CN)_2_^−^]_2_ dimers^20^ and trimers^21^ was identified by the detection of coherences originating from the Au–Au stretching mode.

In their seminal work, Paulus *et al.*, used the insights gained from vibrational coherences to synthetically modify an Fe^ii^ complex to restrict the reaction coordinate for ISC from the metal-to-ligand charge-transfer (MLCT) state, and found that the MLCT state lifetime increased 20-fold.^16^ Similarly, synthetic modification of binuclear Pt^ii^ complexes resulted in two branched ISC processes revealed through analysis of vibrational wavepackets.^22,23^ Vibrational coherences have also been studied for their role in molecular fragmentation,^24^ charge transfer,^25^ and electron transfer processes.^26–29^ In this work, we have studied vibrational coherences in high-spin Mn^iii^ ions, which have a t_2g_^3^e_g_^1^ electron configuration and exhibit axial JT distortion,^30^ with the aim to synthetically control the wavepacket motion in the excited state. In our previous work, it was shown that ultrafast excitation of ligand-field transitions in Mn(acac)_3_ (acac = acetylacetone) and the SMM [Mn^iii^_3_O(Et-sao)_3_(β-pic)_3_(ClO_4_)] (Mn_3_) generated a vibrational wavepacket on the excited state potential energy surface, causing a switch in the JT distortion from axial to equatorial.^14^ For Mn(acac)_3_ this causes a switch from axially elongated to compressed JT distortion due to the transition of electron density from the antibonding d_*z*^2^_ orbital into the antibonding d_*x*^2^−*y*^2^_ orbital. This switch is expected to change the magnitude and direction of the magnetic anisotropy, which could provide a method to optical control their magnetic properties. In Mn_3_, the JT axis lies perpendicular to the plane formed by the three Mn^III^ ions, which are arranged in a triangle. We found that in Mn_3_, equatorial expansion was not possible due to the strong bonds in the plane of the triangle provided by the μ_3_-oxo and μ-oxime bridging ligands. The main motion of the wavepacket was the displacement of the three Mn ions along the *z*-axis, which leads to a modulation of the axial JT distortion and a shorter compressed-state lifetime. We similarly studied Mn^III^(terpy)(X_3_) (X = F, Cl, N_3_), where the terpyridine ligand restricts the JT axis, and found that the main reaction coordinate for the photoinduced JT switch was a single pincer-like mode.^31^ These initial insights are promising, and suggest that the wavepacket can be constrained in the excited state by designing molecules such that certain geometrical pathways are preferred.

To further our understanding about how structural perturbations can affect the lifetime of the compressed JT-state and steer wavepacket motion along excited state potential energy surfaces (PES), we have investigated the incorporation of the 1,4,8,11-tetraazacyclotetradecane (cyclam) ligand to restrict the equatorial bonds in Mn^III^ monomers. Using ultrafast TAS we have studied the photoinduced dynamics initiated by excitation of the lowest ligand-field transition of [Mn(cyclam)(H_2_O)_2_]^3+^. We found vibrational wavepackets were launched along the excited state PES, revealing a complex reaction coordinate to form the axially compressed excited state. The compressed-state lifetime was found to be several-orders of magnitude shorter than for Mn(acac)_3_.^14^

## 2. Method

**Fig. 1:**
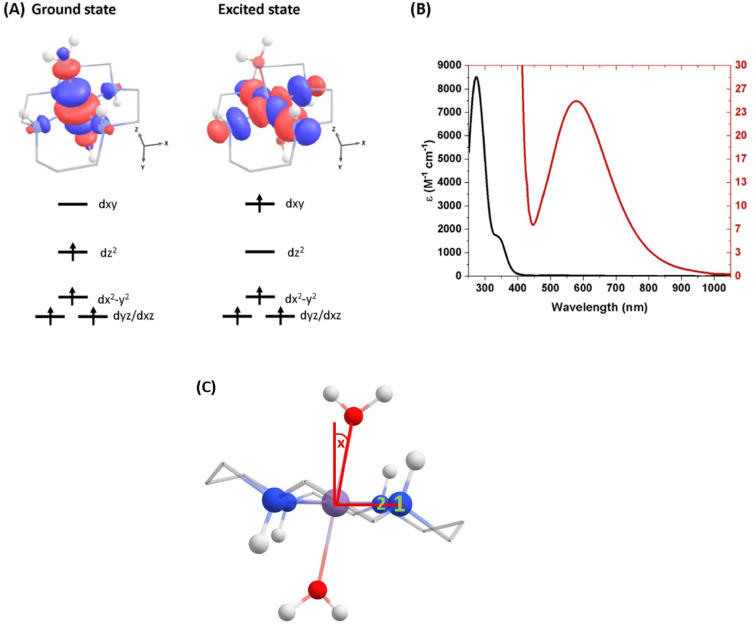
(A) Ground-state structure and electron configuration of the ground state and first excited quintet state of [Mn(cyclam)(H_2_O)_2_]^3+^. Molecular orbitals are shown for the d_*xy*_ ← d_*z*^2^_ transition from the NEVPT2 calculations. The principal *C*_2_ axis lies between the equatorial bonds (see Fig. S10 of the ESI†), meaning the d_*xy*_ orbital lies along the nitrogen bonding axis and the d_*x*^2^−*y*^2^_ is a non-bonding orbital. Hydrogen atoms on carbons have been omitted for clarity. (B) UV-vis of a 1 mM (black) and 30 mM (red) solution of [Mn(cyclam)(H_2_O)_2_]^3+^ in water. (C) Definition of the axial tilt angle *x*, which defines the deviation from 90° between O–Mn–N^*x*^ (*x* = 1 or 2 and numbered in green). Manganese (purple), nitrogen (blue), oxygen (red), carbon (grey), hydrogen (white).

The photoinduced dynamics initiated by excitation of the lowest ligand-field electronic transition of [Mn(cyclam)(H_2_O)_2_]^3+^ were studied using TAS with a broadband white-light probe. Spectra were collected following the 640 nm photoexcitation of 30 mM [Mn^III^(cyclam)(H_2_O)(OTf)][OTf]_2_ in water (Fisher scientific, HPLC grade). [Mn^III^(cyclam)(H_2_O)(OTf)][OTf]_2_ was synthesised following a modified procedure previously reported,^30^ and outlined in Section 3 of the ESI.† Because our samples use water as a solvent we assume the axial OTf ligand to be readily replaced with water. This is confirmed by our steady state UV-vis spectra which are shown in Fig. 1 and match previous reports for [Mn^III^(cyclam)(H_2_O)_2_][OTf]_3_ in water.^30^ Complementary experiments were conducted using 30 mM [Mn^III^(cyclam)Cl_2_][Cl] in 4 M HCl_(aq)_ and methanol with a excitation wavelength of 770 and 920 nm respectively, and shown in the ESI.† Raman spectra were measured using a Renishaw Raman microscope with a laser wavelength of 514 nm.

### 2.1 Transient absorption spectroscopy

The TA instrumental set-up has previously been descried elsewhere,^31^ and only important experimental details are reported here. Samples were flowed at a rate of 16 μL min^−1^ through a Starna flow cuvette with a 0.2 mm pathlength. Samples were intersected by pump pulses of 40 fs pulse duration and 10 mJ cm^−2^ fluence produced by the 1028 nm pumped optical parametric amplifier (Orpheus F, Light conversion) running at a repetition rate of 1 kHz. High fluences were used due to the low absorption of the ligand-field transitions of these complexes, but the signal as a function of pump power shows we are still in the single-photon regime (see Fig. S8 of the ESI†). After a delay of up to 3 ns, controlled by an optical delay line, the excited samples were probed by a weak broadband white-light probe generated in a 1028 nm pumped CaF_2_ plate. We achieve a temporal cross-correlation in the pure solvent of ≤180 fs across the probe spectral range, although the maximum frequency of the detected wavepacket is determined by the duration of the pump pulse.^32^

A mechanical chopper intercepted the pump pulse at a repetition rate of 500 Hz to produce pumped and unpumped spectra so that a pump-induced difference spectra could be derived. The pump pulse was focussed on the sample using a spherical concave mirror to a beam radius of 100 μm at 1/*e*^2^ of the intensity. The excited samples were then probed by spatially overlapping and focusing a broadband probe with a wavelength range of 320–800 nm to 50 μm using a spherical concave mirror. The relative polarisation direction of the pump and probe was set to magic angle (54.7°) and the intercept angle between the pump and probe was 1°.

After the sample, the probe was collimated and dispersed by a prism onto a fast charge coupled device (CCD) camera (Entwicklungsbuero Stresing) equipped with a Hamamatsu S7031-0907 sensor with 512 × 58 active pixels. A portion of the white-light beam was split from the probe beam path before the sample and passed to a matched spectrometer to obtain a reference spectrum of each probe pulse. This reference was used in the processing of the TAS data to reduce the shot-to-shot noise in the transient spectra.

### 2.2 Computational details

#### 2.2.1 Ground state

Geometry optimisations and vibrational frequencies of [Mn(cyclam)(H_2_O)_2_]^3+^ in its ground axially elongated quintet state (Q_0_-elongated) were carried out using the Gaussian 16 quantum chemistry package.^33^ The cyclam ligand is assumed to adopt the *trans*-III conformation (Fig. 1(C)).^30^ Ground state computations were optimised using the PBE0 functional and the Def-2-SVP basis set with no symmetry restraints. No imaginary frequencies were found, confirming that geometries are at a minimum of the potential energy surface. Because we perform our experiments in water, we have also computed the structure of the complex with 4 explicit water molecules to account for hydrogen-bonding interactions. However, computations regarding the nature of the excited state are similar, and for clarity, we illustrate our findings using the gas-phase structure.

#### 2.2.2 Excited state

Multi-configurational calculations were calculated using the ORCA 5.0 computational package.^34^ The electronic structure of [Mn(cyclam)(H_2_O)_2_]^3+^ was studied using PBE0 geometries and CASSCF/NEVPT2 energies. An active space was selected consisting of 8 electrons in 7 orbitals with the Def-2-SVP basis set. The active space took into account the metal-centred antibonding d-orbitals and bonding d_*z*^2^_ and d_*xy*_ orbitals involving lone pairs on the aqua and cyclam ligands (see Fig. S10 of the ESI†). Inclusion of additional molecular orbitals involving the aqua and cyclam lone pairs made no considerable improvement to the computations (see Table S1 of the ESI†). The RIJCOSX approximation for integral calculations was employed to speed up the calculations.^35^ Results have been compared to TDDFT computed with the G16 computational package using the PBE0 functional with the Def-2-SVP basis set.^33^ Summary of the CASSCF/NEVPT2 transition energies are shown in Table 2. TDDFT well-reproduced CASSCF/NEVTPT2 vertical excitation energy to within 0.2 eV and orbital transitions for the first excited quintet state (Q_1_) owing to its highly single reference nature (see ESI†). On this basis, we have computed the geometry and vibrational frequencies for the Q_1_ state using TDDFT with the PBE0 functional and Def-2-SVP to guide our assignment of vibrational coherences. We have performed these calculations for [Mn(cyclam)(H_2_O)_2_]^3+^ without consideration of hydrogen-bonding interactions because of convergence problems. Although we expect the axial bond length to significantly shorten with these interactions, our vibrational frequency analysis in its ground state indicate the axial stretching frequency to be similar (within 35 cm^−1^) and therefore assume our excited state vibrational frequencies to be of a reasonable prediction. The geometry of a position close to the conical intersection between the Q_0_ and Q_1_ states was optimised using TDDFT at the PBE0/Def-2-SVP level of theory on the ORCA 5.0 computational package.^34^

## 3. Results and discussion

### 3.1 TA experiments of [Mn(cyclam)(H_2_O)_2_]^3+^

The steady-state UV-vis spectrum of [Mn(cyclam)(H_2_O)_2_]^3+^ in water (Fig. 1(B)) shows a single weak but broad band with an extinction coefficient of 25 M^−1^ cm^−1^ around 580 nm, which we assign to the lowest ligand field d_*xy*_ ← d_*z*^2^_ transition based on our electronic structure calculations (Table 2). Other ligand-field transitions are predicted below 400 nm and are likely masked by intense charge-transfer bands close by, which agrees well with our observation of only one weak band in the visible region and intense transitions below 370 nm (Fig. 1(B)). Our calculations show that the axial bond lengths of [Mn(cyclam)(H_2_O)_2_]^3+^ are shortened by hydrogen-bonding, reducing the energy of the d_*xy*_ ← d_*z*^2^_ transition (Table 2). For this reason, we attribute the broadness of the d_*xy*_ ← d_*z*^2^_ band to be caused by hydrogen-bonding interactions of solvent water molecules with the complex. Our findings are consistent with crystallographic data, which show the axial bond length to be different for different hydrogen-bonded conformers with OTf anions.^30^

**Fig. 2:**
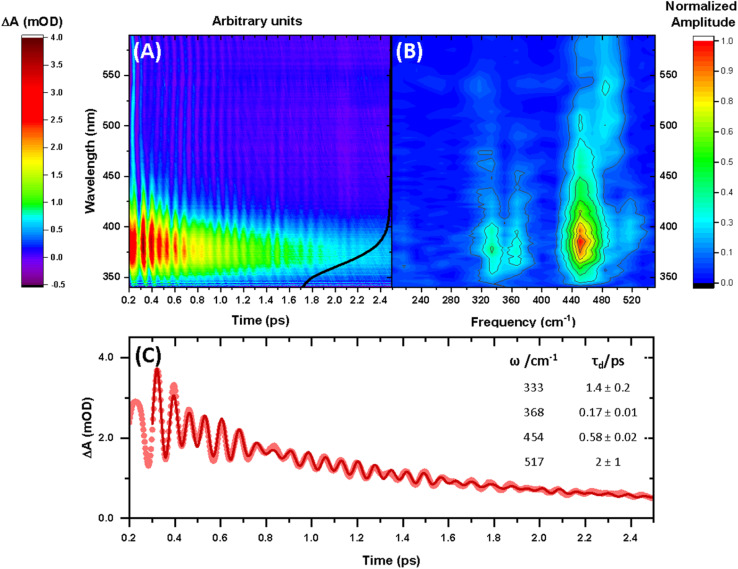
(A) Transient absorption spectra obtained between 340 and 590 nm after 640 nm photoexcitation of [Mn(cyclam)(H_2_O)_2_]^3+^. The black curve shows the ground state UV-vis spectra (arbitrary intensity). (B) Fast Fourier transform (FFT) of the residuals extracted from biexponential kinetic fits across the probe spectral range. (C) Kinetic trace and fit of the excited state absorption taken as an average between 370–380 nm. Dots are experimental data points, and the red curve is a sum of the kinetic fit to a biexponential decay (*τ*_1_ = 0.9 ± 0.1 ps and *τ*_2_ = 3.7 ± 0.1 ps) and exponentially dampened cosine functions with frequencies (*ω*) and dephasing times (*τ*_d_) (numerical results summarised in the inset). A background frequency of 487 cm^−1^ originating from the cuvette was fit to a fixed dephasing time of *τ*_B_ = 0.6 ps, which was determined by fitting an exponentially dampened cosine function to a kinetic trace without sample (see Fig. S6 in the ESI†).

**Fig. 3:**
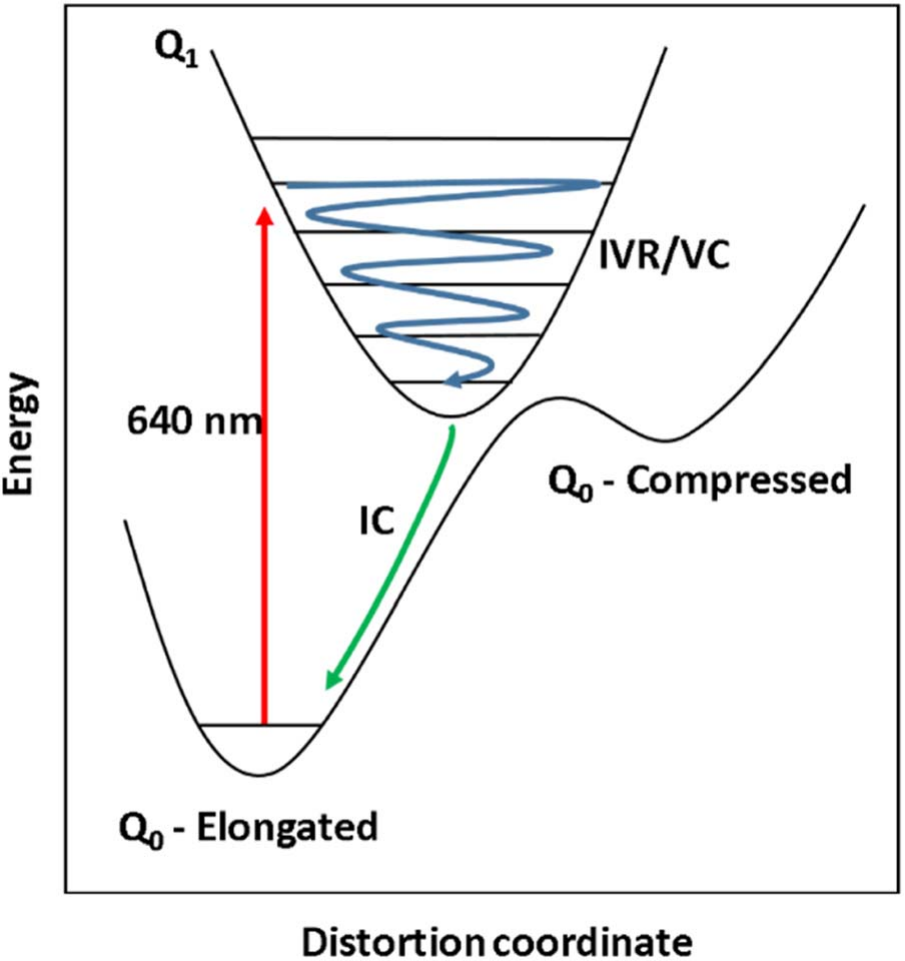
Schematic representation of the potential energy surfaces of the ground and first excited state (black curves) of a pseudo JT-distorted complex. Excitation at 640 nm (red arrow) causes excitation to the Q_1_ state, generating vibrational wavepackets (blue arrow). The Q_1_ state undergoes intramolecular vibrational energy redistribution (IVR) and vibrational cooling (VC) before subsequent internal conversion (IC) back to the Q_0_ – elongated state.

The 640 nm photoexcitation of 30 mM [Mn(cyclam)(H_2_O)_2_]^3+^ in water results in the TA spectra shown in Fig. 2(A). A Fourier analysis of the residuals extracted from the kinetic traces as a function of probe wavelength is shown in Fig. 2(B). Solutions of [Mn(cyclam)Cl_2_][Cl] in 4 M HCl_(aq)_ and in methanol have also been studied and are shown in the ESI.† The TA spectra in Fig. 2(A) reveal a positive feature at 375 nm that forms within the instrument response function (IRF) (<180 fs), and which we assign to a signature of the Q_1_ state on the basis that we photoexcite the lowest ligand-field transition of [Mn(cyclam)(H_2_O)_2_]^3+^. The band subsequently decays with biexponential time constants of *τ*_1_ = 0.9 ± 0.1 ps and *τ*_2_ = 3.7 ± 0.1 ps. The kinetic trace and fitting are shown in Fig. 2(C). The much shorter lifetime of the compressed-state than our previous findings for Mn(acac)_3_ (ref. 14) is likely due to the restriction of the equatorial axis disfavouring equatorial elongation, and is further discussed in Section 3.2. We find no evidence for ground state bleach (GSB) features despite strong bands expected below 400 nm, which is likely due to strong overlap from the excited state absorption signal. The ground state absorption signatures around 580 nm in the steady state UV-vis spectrum are likely too weak to be observed in the TA spectra. We assign the shorter time constant *τ*_1_ to encompass contributions from cooling mechanisms such as intramolecular vibrational energy redistribution (IVR) and dissipation of excess energy to the solvent bath. This is made evident by the shift to shorter wavelengths and narrowing of the excited state absorption signature over the first 1 ps. We attribute the longer time constant *τ*_2_ to internal conversion back to the elongated Q_0_ state. We discount branching into the compressed Q_0_ state on the basis that we do not observe a GSB feature at time durations >5 ps (see Fig. S7 of the ESI†), instead suggesting a reformation of the elongated Q_0_ state. It is possible the compressed Q_0_ state is formed within our IRF (<180 fs), and instead the decay of the band at 375 nm could be attributed to the compressed Q_0_ state reforming the elongated structure. But we prefer assignment to the Q_1_ state because frequency analysis of our TDDFT computations near the point of the conical intersection reveals one imaginary frequency (Fig. S12 of the ESI†), encompassing asymmetric stretching motion of the equatorial bonds. Because the asymmetric mode is not involved in the reaction coordinate, we argue that passage through the conical intersection to the compressed Q_0_ state would be much slower than to occur within our IRF (Fig. 3).

**Fig. 4:**
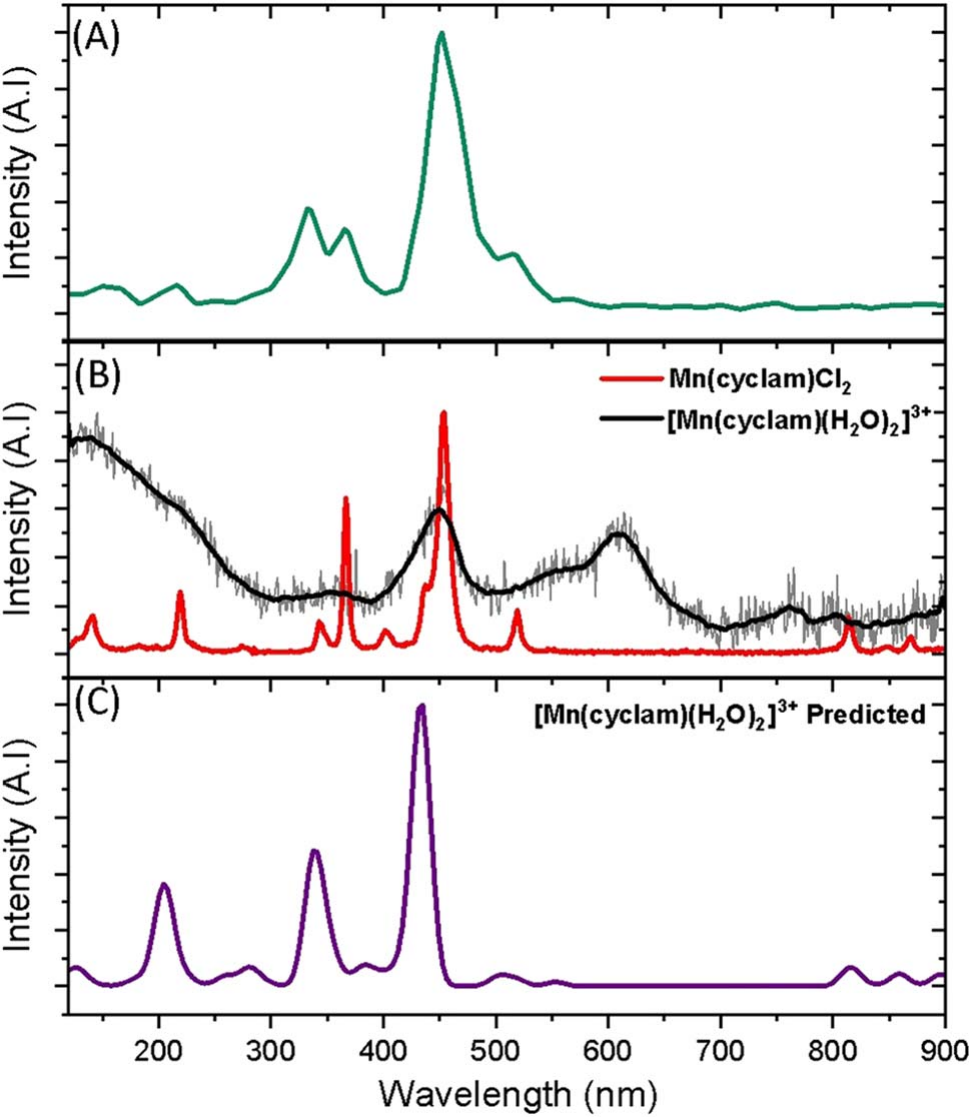
(A) FFT from the residuals extracted at 390 nm from the photoexcitation of [Mn(cyclam)(H_2_O)_2_]^3+^ (TA spectra shown in Fig. 2). (B) Experimental ground state Raman spectra of Mn(cyclam)Cl_2_ crystals (red) and [Mn(cyclam)(H_2_O)]^3+^ in water (grey, black curve – smoothed). (C) Predicted ground state Raman intensities of [Mn(cyclam)(H_2_O)_2_]^3+^.

Superimposed onto the kinetic traces of the excited state absorption signature for [Mn(cyclam)(H_2_O)_2_]^3+^ are oscillations that we attribute to the generation of vibrational wavepackets (Fig. 2(B)). Oscillations are observed in the TA data because the vibrational motion causes a modulation of the extinction coefficient. The frequency at 487 cm^−1^ is a response from the cuvette (see Fig. S5 in the ESI†). To discount the assignment of ground state coherences we have compared the observed frequencies in our TA data to the ground state Raman spectrum of [Mn(cyclam)(H_2_O)_2_]^3+^ as shown in Fig. 4. Excited state coherences can usually be assigned on the basis that observed frequencies appear shifted relative to the ground state Raman spectrum, but because we expect equatorial bonds in the excited state to be of similar frequency we predict that only frequencies corresponding to axial motion will be shifted. To aid in the assignment of the ground state Raman spectrum we have compared the spectrum of [Mn(cyclam)(H_2_O)_2_]^3+^ to [Mn(cyclam)Cl_2_]Cl (Fig. 4(B)). Raman-active bands associated with the motion of the equatorial ligand should be present in both spectra, but bands assigned to axial motion should be different. Our computed Raman spectrum of [Mn(cyclam)(H_2_O)_2_]^3+^ (Fig. 4(C)) agrees well with experiment, and shows the Raman spectrum to be dominated by cyclam modes. This is further supported by our Raman spectrum of [Mn(cyclam)Cl_2_]Cl which is very similar and shows no additional resolvable Raman-active bands below 900 cm^−1^. The band around 610 cm^−1^ for [Mn(cyclam)(H_2_O)_2_]^3+^ likely arises from the triflate counter anion which is expected close by.^36^ In [Mn(cyclam)(H_2_O)_2_]^3+^ axial stretching frequencies are expected around 213 and 227 cm^−1^, but are overlapped by intense frequencies from the cyclam ligand. However, our coherences reveal a frequency at 333 cm^−1^ (Fig. 4(A)) which we assign to axial compression (see Section 3.2) and which is not observed in our ground state Raman spectrum, suggesting an excited state coherence. Assignment to an excited state coherence is further supported by the amplitude of the oscillations being largest at the peak of the excited state absorption signal (see Fig. S9 of the ESI†).

**Fig. 5:**
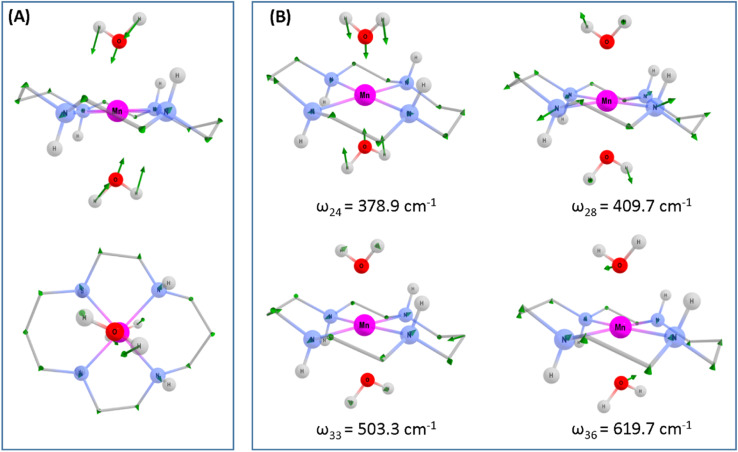
Computed structural displacements and vibrational frequencies of the Q_1_ state of [Mn(cyclam)(H_2_O)_2_]^3+^ at the PBE0/Def-2-SVP level of theory. (A) Ground state geometry of [Mn(cyclam)(H_2_O)_2_]^3+^. Green arrows show the displacement to form the relaxed Q_1_ state geometry. The displacement has been scaled by *a* factor of 2 for clarity. (B) Computed assigned vibrational frequencies for the Q_1_ state of [Mn(cyclam)(H_2_O)_2_]^3+^. Hydrogen atoms on carbons have been omitted. For frequency *ω*_36_ the largest displacement is found for the hydrogen atoms on the axial water ligands. These have been omitted to highlight displacements to other atoms. Manganese (purple), nitrogen (blue), oxygen (red), carbon (grey), hydrogen (white).

We propose that the wavepacket arises from population on the displacive potential energy surface of the excited state caused by a transition of an electron from the d_*z*^2^_ orbital into the d_*xy*_ orbital (Fig. 1). These orbitals are anti-bonding along the bonding axes, which causes a shortening of the axial bonds and a small lengthening of the equatorial bonds, as illustrated in Fig. 5(A). As such, the JT distortion switches from axially elongated, to compressed in the excited state. The frequencies at 333, 368, 454 and 517 cm^−1^ decay in amplitude with time constants of *τ*_d_ = 1.4 ± 0.2, 0.17 ± 0.01, 0.58 ± 0.02 and 2 ± 1 ps, respectively.

### 3.2 Assignment of vibrational coherences

To gain deeper insights into the motions involved in the reaction coordinates and aid the assignment of the vibrational coherences observed in our TA spectra, we have optimised the Q_1_ state of [Mn(cyclam)(H_2_O)_2_]^3+^ in the gas phase, which reveals a minimum on the Q_1_ potential energy surface with a compressed geometry. The predicted changes for the relaxed Q_1_ state compared to the Franck–Condon geometry is illustrated in Fig. 4(A), and values reported in Table 1. Computed harmonic frequencies are further used to support our assignments and are summarised in Fig. 4(B). Our computations suggest a 0.35 Å shortening of the axial bonds, and a 0.07 Å elongation of the equatorial bonds in the gas-phase between the Q_0_-elongated and the relaxed Q_1_ states. The much smaller change in the equatorial bond lengths compared to the axial compression suggests equatorial elongation is disfavoured because of the rigid cyclam ligand, which is one of the main results of our study. Additional geometry changes are predicted in the excited state (Table 1) and discussed below.

**Table 1:** Computed geometries of the inner coordination sphere of [Mn(cyclam)(H_2_O)_2_]^3+^ in the gas phase and a hydrogen-bonded structure using the PBE0 functional with the Def-2-SVP basis set

	[Mn(cyclam)(H_2_O)_2_]^3+^	[Mn(cyclam)(H_2_O)_2_]^3+^ (hydrogen-bonded)
**Ground state (Q**_**0**_ **– elongated)**		
Mn–X/Å	2.328	2.204
Mn–N_avg_/Å	2.070	2.070
Tilt 1/deg^a^	+0.78	+2.12
Tilt 2/deg^a^	+2.82	+2.94
N_4_/deg^b^	0.65	0.11
Symmetry	*C*_2_	*C*_2_

First **excited state (Q**_**1**_**)**		
Mn–X/Å	1.982	
Mn–N_avg_/Å	2.162	
Tilt 1/deg^a^	−0.32	
Tilt 2/deg^a^	+0.20	
N_4_/deg^b^	1.78	
Symmetry	*C*_2_	

**Conical intersection (Q**_**0**_ **/Q**_**1**_ **)**		
Mn–X/Å	1.867	
Mn–N_avg_/Å	2.195	
Tilt 1/deg^a^	−1.07	
Tilt 2/deg^a^	−0.78	
N_4_/deg^b^	3.47	
Δ*E*/kJ mol^−1^^c^	18.0	
Symmetry	*C*_2_	

a Tilt defined and numbered in Fig. 1(C).

b Deviation of the equatorial N atoms from planar.

c Compared to the Q_1_ minimum energy geometry.

**Table 2:** Excitation energies of [Mn(cyclam)(H_2_O)_2_]^3+^ at the NEVPT2(8,7) level with the Def-2-SVP basis set

Electron configuration	[Mn(cyclam)(H_2_O)_2_]^3+^/nm	[Mn(cyclam)(H_2_O)_2_]^3+^ (hydrogen bonded)/nm
d^1^_*yz*_d^1^_*xz*_d^1^_*x*^2^−*y*^2^_d^1^_*z*^2^_d^0^_*xy*_		
d^1^_*yz*_d^1^_*xz*_d^1^_*x*^2^−*y*^2^_d^0^_*z*^2^_d^1^_*xy*_	445.6	630.0
d^1^_*yz*_d^1^_*xz*_d^0^_*x*^2^−*y*^2^_d^1^_*z*^2^_d^1^_*xy*_	301.4	318.0
d^0^_*yz*_d^1^_*xz*_d^1^_*x*^2^−*y*^2^_d^1^_*z*^2^_d^1^_*xy*_	286.4	311.7
d^1^_*yz*_d^0^_*xz*_d^0^_*x*^2^−*y*^2^_d^1^_*z*^2^_d^1^_*xy*_	283.6	311.3

To assess whether coherences arise from the axial or equatorial motions we have conducted experiments for the 770 nm photoexcitation of [Mn(cyclam)Cl_2_][Cl] in 4 M HCl_(aq)_ and the 920 nm photoexcitation of [Mn(cyclam)Cl_2_][Cl] in methanol. Because we expect the solvent to displace Cl^−^ ions for this complex we are unable to definitively assign the complexes in solution (see ESI† for further discussion), but we expect the axial bond properties (containing the Cl^−^ ions) to be different because they have different UV-vis spectra. On this basis, we should observe pronounced shifts for frequencies corresponding to axial motions, but similar frequencies for coherences involving the equatorial ligand. In these experiments we observed frequencies around 370 and 455 cm^−1^, but do not observe the 333 and 517 cm^−1^ components observed in [Mn(cyclam)(H_2_O)_2_]^3+^. Therefore, we suggest that the 333 and 517 cm^−1^ frequencies arise from the modulation of the axial bonds, which is also in agreement with the discussion above on the Raman spectra. More specifically, we suggest the 333 cm^−1^ component to originate from the stretching frequency, *ω*_24_ (predicted at 379 cm^−1^) and the 517 cm^−1^ component to be caused by the see-saw frequency, *ω*_36_ (predicted at 620 cm^−1^), which involves the modulation of the axial tilt angles of [Mn(cyclam)(H_2_O)_2_]^3+^. Frequencies *ω*_34_ and *ω*_35_ are expected around 525 and 539 cm^−1^ but encompass asymmetric motions of the equatorial ligand, which we do not expect to be involved in the reaction coordinate (see Fig. S11 of the ESI†). The modulation of the axial tilt is predicted by our TDDFT geometry optimised structure (Table 1 and Fig. 4(A)), and we suggest is caused by mixing of the d_*z*^2^_ and d_*xz*_ orbitals resulting in a tilted d_*z*^2^_ orbital (Table S2 of the ESI†). Depopulation of the tilted d_*z*^2^_ orbital subsequently removes the axial tilt in the excited state (Table 1). Because the ∼370 and ∼455 cm^−1^ components are observed in all the complexes, and are similar we expect these coherences to arise from the equatorial ligand. From our computations we expect the symmetric equatorial stretching frequencies around 410 (*ω*_28_) and 503 (*ω*_33_) cm^−1^, which agrees well with the experimental observations. Although these frequencies encompass symmetric expansion of Mn–N bonds, the N-atoms break planarity (Table 1) and considerable readjustment of the cyclam ligand occurs. The breaking of the N-atom plane can be rationalised in terms of the mixing of the d_*xy*_ orbital with the d_*yz*_ (see Table S2 of the ESI†). The shorter dephasing time of the coherences originating from the cyclam ligand could be due to rapid IVR as the energy is dissipated into the ligand backbone. Multiple frequencies suggest a complex reaction coordinate, contrasting findings for Mn_3_ and Mn(terpy)X_3_ (X = F, Cl, N_3_) complexes, where restriction of the complexes only resulted in coherent oscillations from a single normal mode.^14,31^

## 4. Conclusion

Photoexcitation of the lowest ligand-field transition of [Mn(cyclam)(H_2_O)_2_]^3+^ (d_*xy*_ ← d_*z*^2^_) results in a switch from the axially elongated Jahn–Teller distortion present in the Q_0_ ground-state (d^1^_*yz*_d^1^_*xz*_d^1^_*x*^2^−*y*^2^_d^1^_*z*^2^_d^0^_*xy*_) to that in the Q_1_ excited-state (d^1^_*yz*_d^1^_*xz*_d^1^_*x*^2^−*y*^2^_d^0^_*z*^2^_d^1^_*xy*_) which is axially compressed. The dynamics observed are a result of an electron being promoted from the axial-antibonding d_*z*^2^_ orbital into the equatorial-antibonding d_*xy*_ orbital. This in turn causing a shortening of the axial bonds and an elongation of the equatorial bonds. The Q_1_ excited-state lifetime is several orders of magnitude shorter than our previous findings for the excited-state in Mn(acac)_3_ (ref. 14) due to the restrictive cyclam ligand disfavouring equatorial expansion. We have observed oscillations in the femtosecond transient absorption spectra at 333, 368, 454 and 517 cm^−1^, which we have attributed to an excited state vibrational coherence. Multiple frequencies suggest a complex reaction coordinate. In combination with quantum chemical electronic structure calculations, we have identified the main reaction coordinate as a switch from axial elongation to compression, but also observe modulations of the axial tilt angle. These results provide insights into the consequence of restricting the equatorial axis to the excited state dynamics of Mn^iii^ octahedral complexes, and provides valuable information which can be used to develop synthetic strategies for the optical control of magnetic anisotropy of single-molecule magnets.

## Author contributions

R. P. performed the transient absorption experiments, computations, analysed the data, and wrote the manuscript. A. E. B. performed the synthesis, characterisation, wrote the synthetic methodology and reviewed the manuscript. E. K. B. and J. O. J. conceptualized the experiments, acquired funding, and edited the manuscript.

## Conflicts of interest

There are no conflicts to declare.

## Supplementary Material

SC-014-D3SC01506H-s001
