# Supplementary material for: Equatorial restriction of the photoinduced Jahn–Teller switch in Mn(iii)-cyclam complexes
Source: Chem Sci. 2023 May 26;14(24):6621–30. doi: 10.1039/d3sc01506h (PMC10284123; doi:10.1039/d3sc01506h)
Supplement: SC-014-D3SC01506H-s001 [file SC-014-D3SC01506H-s001.pdf]

Electronic Supplementary Material (ESI) for Chemical Science.
This journal is © The Royal Society of Chemistry 2023

Supplementary Information (SI) for: Equatorial restriction of the photo-induced Jahn-Teller switch in Mn(III)-cyclam complexes

Ryan Phelps,[a] Alvaro Etcheverry-Berrios,[a] Euan K. Brechin[a] and J. Olof Johansson[*,a]

Contents

Section 1 – Spectroscopy 2
Steady state UV-Vis *2*
Transient absorption spectroscopy *3*
Section 2 – Computational methods 7
Section 3 – Synthesis and characterisation 11
Methods *11*
Mass Spectrometry 11
IR Spectrometry 11
CHN Analysis 11
Single Crystal X-Ray Diffraction 11
Powder X-Ray Diffraction 11
Synthesis *12*
Synthesis of $[Mn^{III}(cyclam)Cl_2][Cl]$ (**1**) 12
Synthesis of $[Mn^{III}(cyclam)(OH_2)(OTf)][OTf]_2$ (**2**) 12
Characterisation *14*
Mass Spectrometry 14
$[Mn^{III}(cyclam)Cl_2][Cl]$ (**1**) 14
$[Mn^{III}(cyclam)(OH_2)(OTf)][OTf]_2$ (**2**) 15
Infrared Spectroscopy 16
$[Mn^{III}(cyclam)Cl_2][Cl]$ (**1**) 16
$[Mn^{III}(cyclam)(OH_2)(OTf)][OTf]_2$ (**2**) 16
Single Crystal X-Ray Diffraction 17
$[Mn^{III}(cyclam)(OH_2)(OTf)][OTf]_2$ (**2**) 17
Powder X-Ray Diffraction 21
$[Mn^{III}(cyclam)Cl_2][Cl]$ (**1**) 21
$[Mn^{III}(cyclam)(OH_2)(OTf)][OTf]_2$ (**2**) 22
References 23

Section 1 – Spectroscopy

Steady state UV-Vis

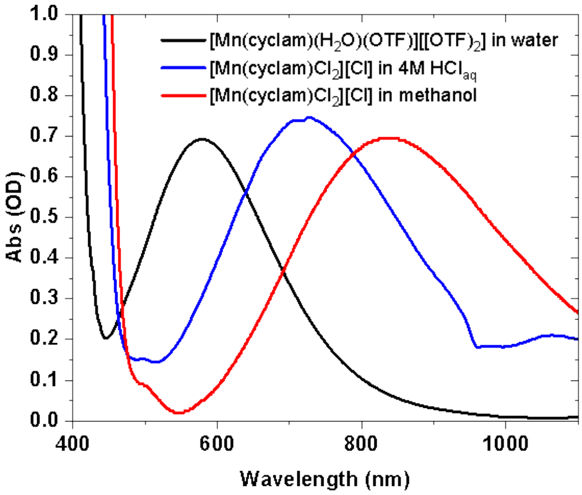

Figure S1 – Steady state UV-Vis spectra of $[Mn(cyclam)(H_2O)(OTf)][OTf]_2$ in water, $[Mn(cyclam)Cl_2][Cl]$ in 4M $HCl_{(aq)}$ and methanol (intensity x5 due to lower solubility).

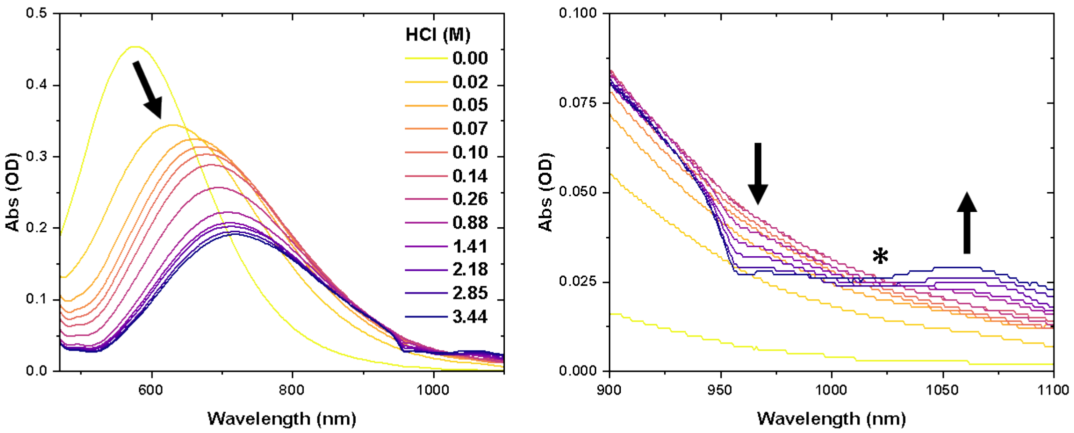

Figure S2 – Steady state UV-Vis spectra of $[Mn(cyclam)(H_2O)_2][OTf]_3$ in water at various concentrations of HCl for probe wavelengths 480-1100 nm (Left) and 900-1100 nm (Right). Due to the expectation of various complexes in equilibrium we are unable to determine extinction coefficients. Black arrows show the direction of change upon addition of HCl. The * shows the position of an isobestic point.

Our computations (see Table S1) suggest the lowest ligand field transition for $[Mn(cyclam)Cl_2]^+$ is expected around 1392 nm with no other ligand-field transitions expected in the visible region. The predicted transition agrees well with the weak feature that appears at 1060 nm for $[Mn(cyclam)(H_2O)(OTf)][OTf]_2$ and $[Mn(cyclam)Cl_2][Cl]$ at higher concentrations of HCl. A feature around 970 nm decreases in intensity at high HCl concentrations which we assume to be $[Mn(cyclam)(H_2O)Cl]^{2+}$ on the basis that our computations show a ~180 nm blue shift relative to $[Mn(cyclam)Cl_2]^+$. The decrease in intensity for higher concentrations is due to the conversion into

$[Mn(cyclam)Cl_2]^+$, where an isobestic point develops around 1025 nm. Because no ligand-field bands are expected in the visible region for either complex baring a Cl ion, the assignment of the broad 700 nm band still remains elusive. We speculate that the shift in the band relative to the $[Mn(cyclam)(H_2O)_2]^{3+}$ at 0 M HCl could be due to either changes in the local solvent environment or from the protonation of the water ligands. Nevertheless, the changes in the position of the band are a reasonable indicator for changes in the axial bond properties.

Transient absorption spectroscopy

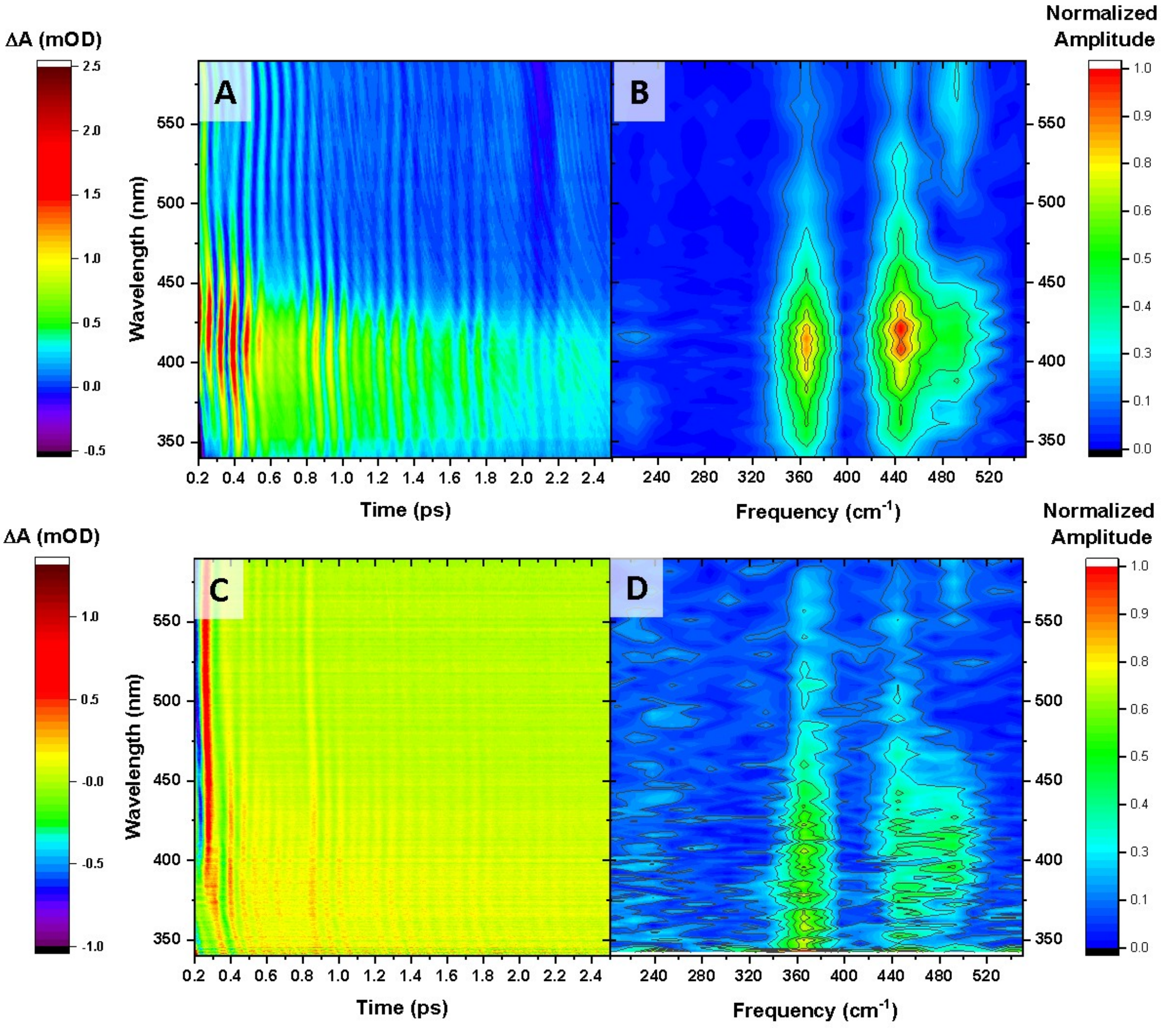

Figure S3 – TA spectra taken for (A) – 770 nm photoexcitation of 30mM $[Mn(cyclam)Cl_2][Cl]$ in 4M $HCl_{(aq)}$ (B) – 920 nm photoexcitation of 30 mM $[Mn(cyclam)Cl_2][Cl]$ in methanol. FFT analysis of the residuals for the spectral probing range are shown in (C) and (D) respectively. The much lower signal for the methanol solutions is due to the low solubility of the complex.

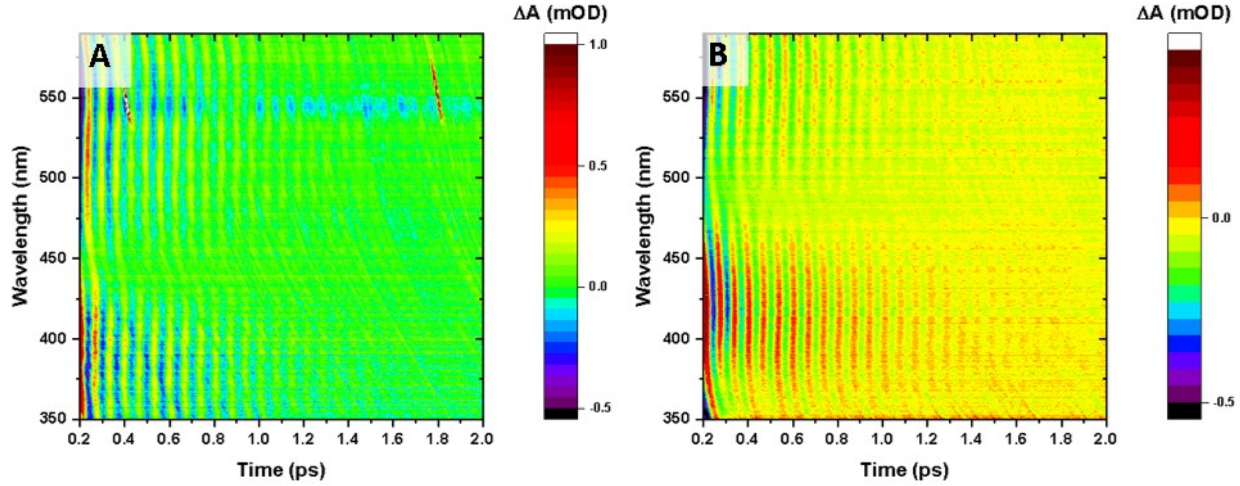

Figure S4 – Background TA experiments in water, in the absence of Mn complexes using pump wavelengths of A – 640 nm and B – 770 nm.

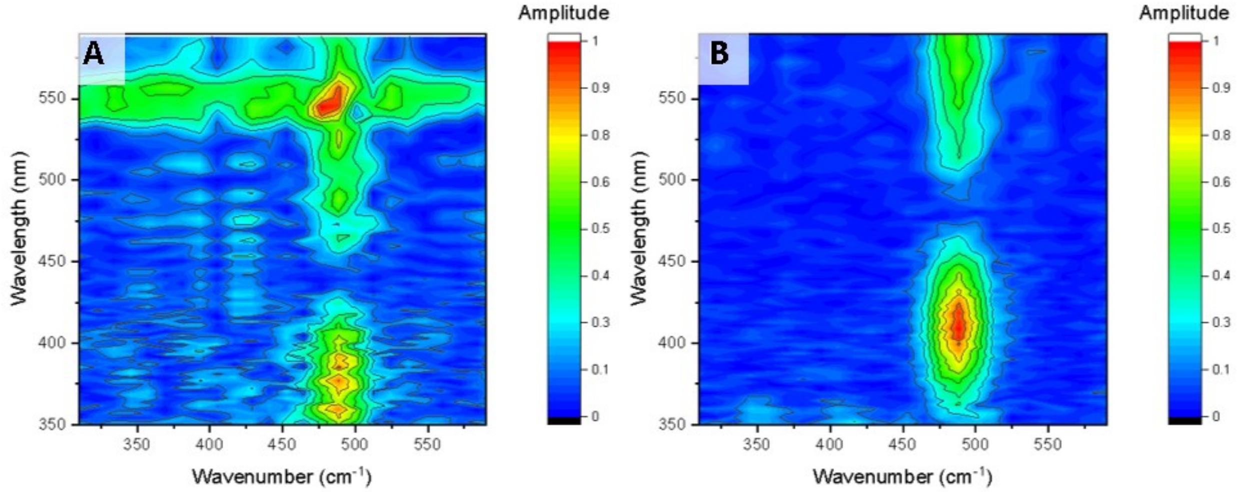

Figure S5 – Background FFT from TA experiments in water, in the absence of Mn complexes using pump wavelengths of A – 640 nm and B – 770 nm.

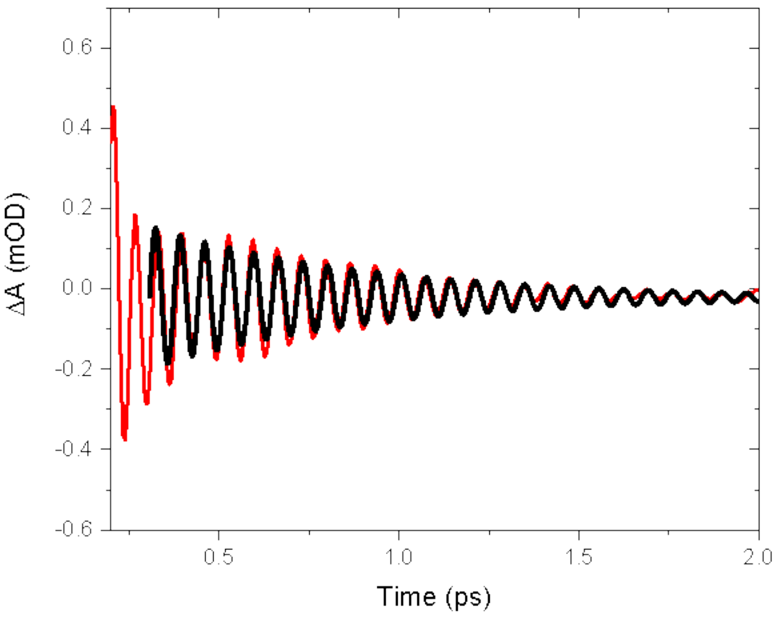

Figure S6 – Kinetic trace taken as an average for probe wavelengths 370-390 nm for the TA measurement conducted using a 640-nm pump of water in the absence of Mn complexes. The red trace shows the experimental data, and the black curve is the fit to an exponentially dampened cosine function. The frequency was fixed to 487 cm^{-1} and fit to dephasing time of 0.60 ± 0.03.

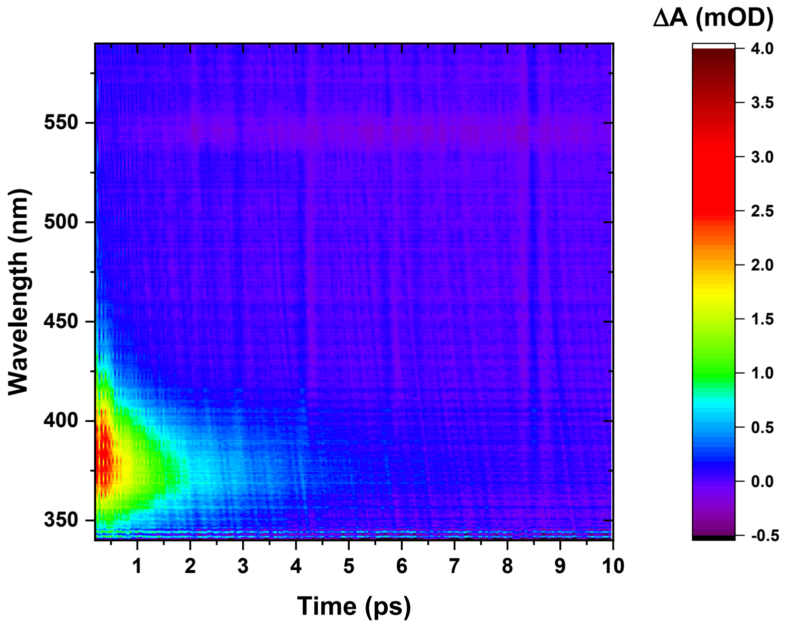

Figure S7 – TA spectra for the 640 nm photoexcitation of $[Mn(cyclam)(H_2O)_2]^{3+}$ in water for time durations between 0.2-10 ps.

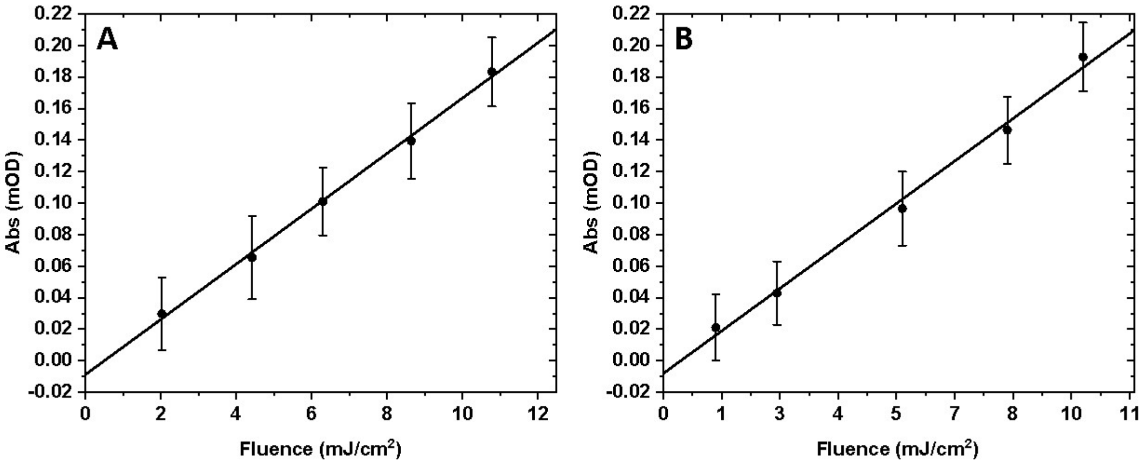

Figure S8 – Pump fluence dependence for A – 30 mM $[Mn(cyclam)(H_2O)(OTf)][OTf]_2$ in water. The average absorption was taken between 350-400 nm at a fixed time delay of 2 ps. B – 30 mM sample of $[Mn(cyclam)Cl_2][Cl]$ in 4M $HCl_{(aq)}$. The average absorption was taken between 360-410 nm at a fixed time delay of 2 ps.

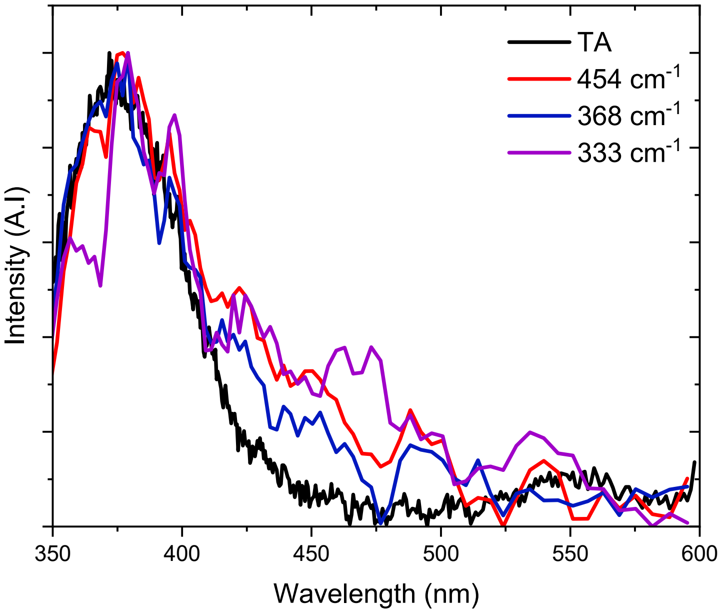

Figure S9 – TA spectrum at 1 ps (black) and oscillation amplitudes for frequencies 454 cm^{-1} (red), 368 cm^{-1} (blue) and 333 cm^{-1} (pruple)

Section 2 – Computational methods

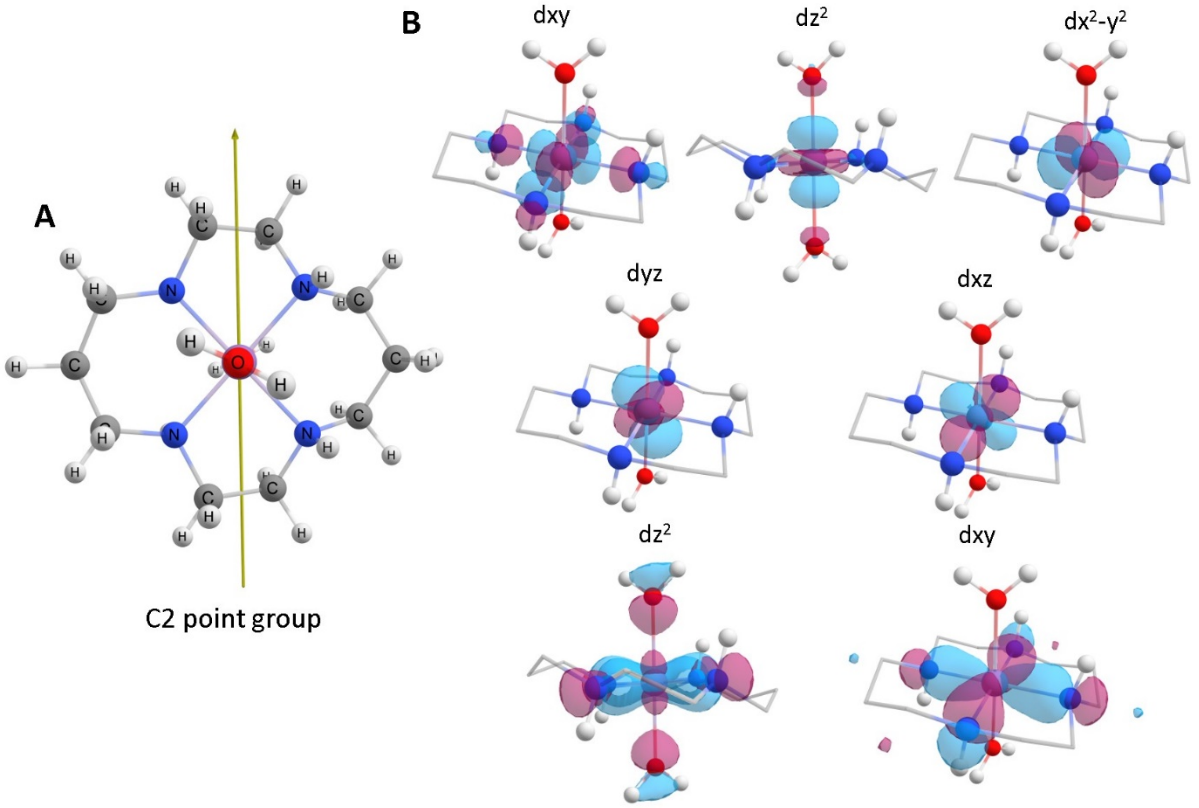

Figure S10 – A – C2 axis of $[Mn(cyclam)(H_2O)_2]^{3+}$. B – Molecular orbitals used in the active space for NEVPT2 computations. Red and blue shows opposite phases to the molecular orbitals.

Table S1 – Predicted UV-Vis spectra of a series of $[Mn(cyclam)X_2]^{n+}$ (X = H_2O, Cl) complexes at the NEVPT2(8,7) level of theory with the Def-2-SVP basis set. The parenthesis show computations with an expanded active space with 12 electrons in 9 orbitals, which also considered additional metal centred bonding d-orbitals. The quintet states are highly single configurational with >0.88 coefficients for the electron configurations reported. The first excited quintet state has a coefficient of 0.95. The triplet states are highly multiconfigurational and typically have no dominant electron configuration.

	$[Mn(cyclam)(H_2O)_2]^{3+}$ / nm	$[Mn(cyclam)(H_2O)_2]^{3+}$ HB / nm	$[Mn(cyclam)Cl_2]^{+}$ / nm	$[Mn(cyclam)(H_2O)Cl]^{2+}$ / nm
		Quintet		
$d_{yz}^1 d_{xz}^1 d_{x^2-y^2}^1 d_{z^2}^1 d_{xy}^0$	-	-	-	-
$d_{yz}^1 d_{xz}^1 d_{x^2-y^2}^1 d_{z^2}^0 d_{xy}^1$	445.6 (445.8)	630.0	1391.9	1225.2
$d_{yz}^1 d_{xz}^1 d_{x^2-y^2}^0 d_{z^2}^1 d_{xy}^1$	301.4 (301.8)	318.0	342.7	337.8
$d_{yz}^0 d_{xz}^1 d_{x^2-y^2}^1 d_{z^2}^1 d_{xy}^1$	286.4 (291.1)	311.7	342.0	337.0
$d_{yz}^1 d_{xz}^0 d_{x^2-y^2}^1 d_{z^2}^1 d_{xy}^1$	283.6 (284.7)	311.3	333.4	336.9
		Triplet		

T_0	594.1	733.6	1163.1	1044.5
T_1	592.9	704.9	1076.3	1041.0
T_2	533.3	678.6	1033.2	998.3
T_3	429.3	437.0	482.2	479.6
T_4	384.6	395.5	425.0	419.6

HB – Computed using 4 explicit water molecules hydrogen bonded to the axial ligands

Table S2 – d-orbital atomic orbital contributions to HOMO and LUMO molecular orbitals.

Atomic orbital	HOMO	LUMO
dz^2	79.0	0.0
dxz	11.4	0.0
dyz	0.0	3.9
dx^2-dy^2	0.4	0.0
dxy	0.0	72.0

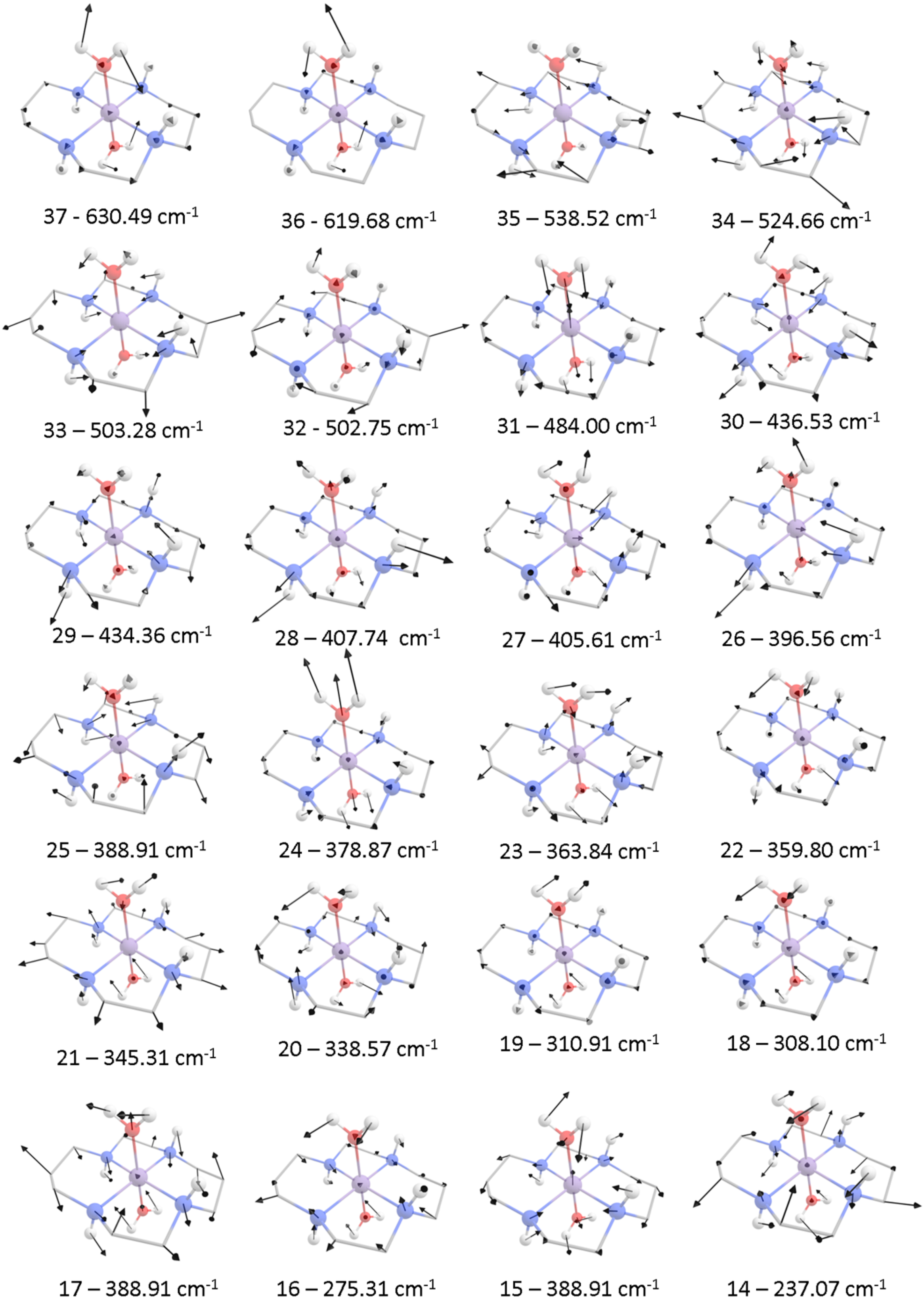

Figure S11 – Computed vibrational frequencies for the first excited quintet state of $[Mn(cyclam)(H_2O)_2]^{3+}$. Values are computed using TDDFT at the PBE0/Def2SVP level of theory.

Manganese (purple), nitrogen (blue), oxygen (red), carbon (grey), hydrogen (white). Black arrows show the displacement vectors.

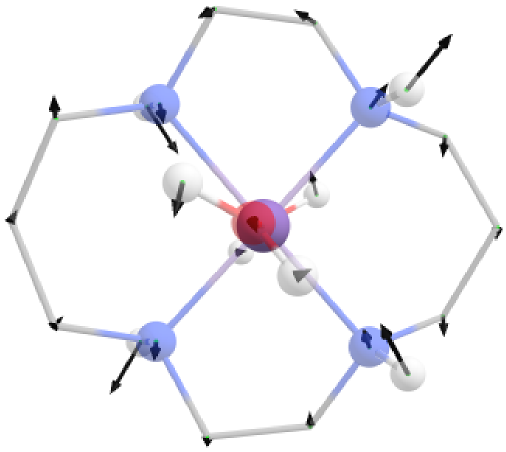

Figure S12 – Computed imaginary vibrational frequency for a position close to the Q_1/Q_0 conical intersection of $[Mn(cyclam)(H_2O)_2]^{3+}$. Values are computed using TDDFT at the PBE0/Def2SVP level of theory. Manganese (purple), nitrogen (blue), oxygen (red), carbon (grey), hydrogen (white). Black arrows show the displacement vectors.

Section 3 – Synthesis and characterisation

Methods

Mass Spectrometry

Electrospray ionisation (ESI) mass spectra were obtained on a Bruker microTOF II instrument. All scans in the experimental data are for positive ions. All analyses were performed with Bruker Compass DataAnalysis, Version 5.0 SR1 and plotted using Origin 2019.

IR Spectrometry

Fourier transform-infrared (ATR FT-IR) measurements were performed on a Perkin Elmer 65 FT-IR spectrometer over the range 3500–550 cm^{-1}.

CHN Analysis

Elemental Analysis for CHN were obtained on a Thermo Fisher Scientific Flash SMART 2000 Elemental Analyser in the School of Geosciences at The University of Edinburgh. Samples were kept at 55 °C for 72 h and freshly ground before analysis.

Single Crystal X-Ray Diffraction

Single crystals of **2** were selected, coated in oil and mounted using a MITIGEN mount on a Bruker SMART APEXII diffractometer equipped with an Oxford Cryosystems Cryostream 700+ low-temperature device, operating at 120.0 K. Cell parameters were retrieved using the CrysAlisPro software[1] and refined using CrysAlisPro The structures were solved using Olex2[2] with the ShelXT[3] structure solution program, using the Intrinsic Phasing solution method. The models were refined with version 2014/7 of ShelXL[4] using Least Squares minimisation. All non-hydrogen atoms were refined anisotropically. Hydrogen atom positions were calculated geometrically and refined using the riding model.

Powder X-Ray Diffraction

Powder XRD measurements were collected on freshly prepared samples of **1** and **2** using a Bruker D8 ADVANCE with nickel filtered Cu radiation at power 40 kW and current 40 mA. Diffraction patterns were measured from 2θ = 2° - 30°, step size 0.0151°.

Synthesis

All reagents were of reagent quality (≥95%), purchased from commercial suppliers (Alfa Aesar, VWR, Fluorochem or Sigma Aldrich) and used without further purification.

Synthesis of [Mn^{III}(cyclam)Cl_2][Cl] (1)

This compound was synthesised following a modified procedure previously reported.[5]

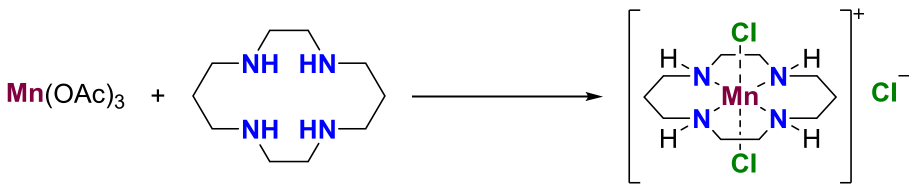

In a 100 mL conical flask, $Mn(OAc)_3\cdot 2H_2O$ (670 mg, 2.50 mmol) was dissolved in 20 mL of MeOH. To this mixture, a cyclam (501 mg, 2.50 mmol) solution in 5 mL of MeOH was added. The resulting solution was stirred at room temperature for 3 h, followed by the addition of 1 mL of HCl_{conc} 12 M, affording a green precipitate. This precipitate was filtered, dissolved in 30 mL of H_2O affording a green solution and treated with 5 mL of HCl_{conc} 12 M, changing its colour to light green. The solution was left undisturbed at room temperature overnight, affording a crystalline green solid. The crystals were collected in a Sartorius filter, washed with a small amount of cold H_2O to afford 656 mg of a crystalline bright green material. Yield: 58%. FTIR: ν/cm^{-1} 3123, 2979, 2853, 1664, 1655, 1648, 1618, 1546, 1438, 1315, 1297, 1127, 1092, 1054, 1022, 1010, 985, 874, 798, 761, 714, 683, 635, 558. MS (ESI): *m/z* calculated for $C_{10}H_{24}MnN_4Cl_2^+$ [M-Cl]$^+$ 325.07487; found 325.07584. Elemental Analysis calculated (%) for $C_{10}H_{24}Cl_3MnN_4\cdot 0.9H_2O$: C, 31.64; H, 6.90; N, 14.76; found: C, 31.90; H, 6.97; N, 14.72.

Synthesis of [Mn^{III}(cyclam)(OH_2)(OTf)][OTf]$_2$ (2)

This compound was synthesised following a modified procedure previously reported.[6]

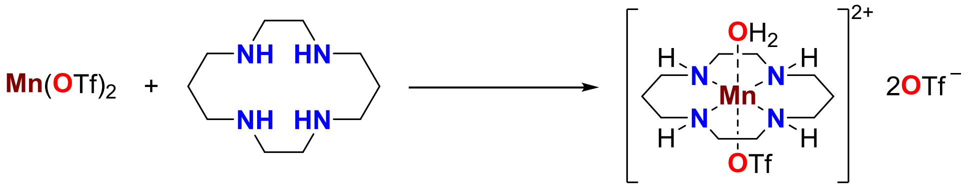

In a 50 mL conical flask, $Mn(OTf)_2$ (2.00 g, 5.66 mmol) was dissolved in 10 mL of EtOH and added to a cyclam (1.13 g, 5.66 mmol) solution in 25 mL of EtOH. The resulting solution was stirred at room temperature for 1 h. After this, the volume was reduced under reduced pressure to ca. 3 mL affording

a dark green precipitate, which was then filtered and dried. This solid was treated with 9 mL of trifluoromethanesulfonic acid 2 M in H_2O and 5 mL of H_2O and stirred at room temperature for 4 h. This resulted in a clear violet solution which was left to slowly evaporate, affording 714 mg of violet crystals which were collected and air-dried. Single crystals for diffraction measurements were obtained in the same batch. Yield: 16%. FTIR: ν/cm^{-1} 3166, 2868, 1677, 1464, 1457, 1432, 1321, 1285, 1260, 1227, 1205, 1178, 1158, 1113, 1088, 1049, 1025, 1014, 934, 904, 880, 803, 766, 758, 730, 628, 576. MS (ESI): *m/z* calculated for $C_{12}H_{24}F_6MnN_4O_6S_2^+$ [M-OTf-H_2O]$^+$ 553.04238; found 553.04086. Elemental Analysis calculated (%) for $C_{13}H_{24}F_9MnN_4O_9S_3\cdot1.5H_2O$: C, 21.40; H, 3.73; N, 7.68; found: C, 21.44, H, 3.77; N, 7.61.

Characterisation

Mass Spectrometry

*[Mn^{III}(cyclam)Cl$_2$][Cl] (**1**)*

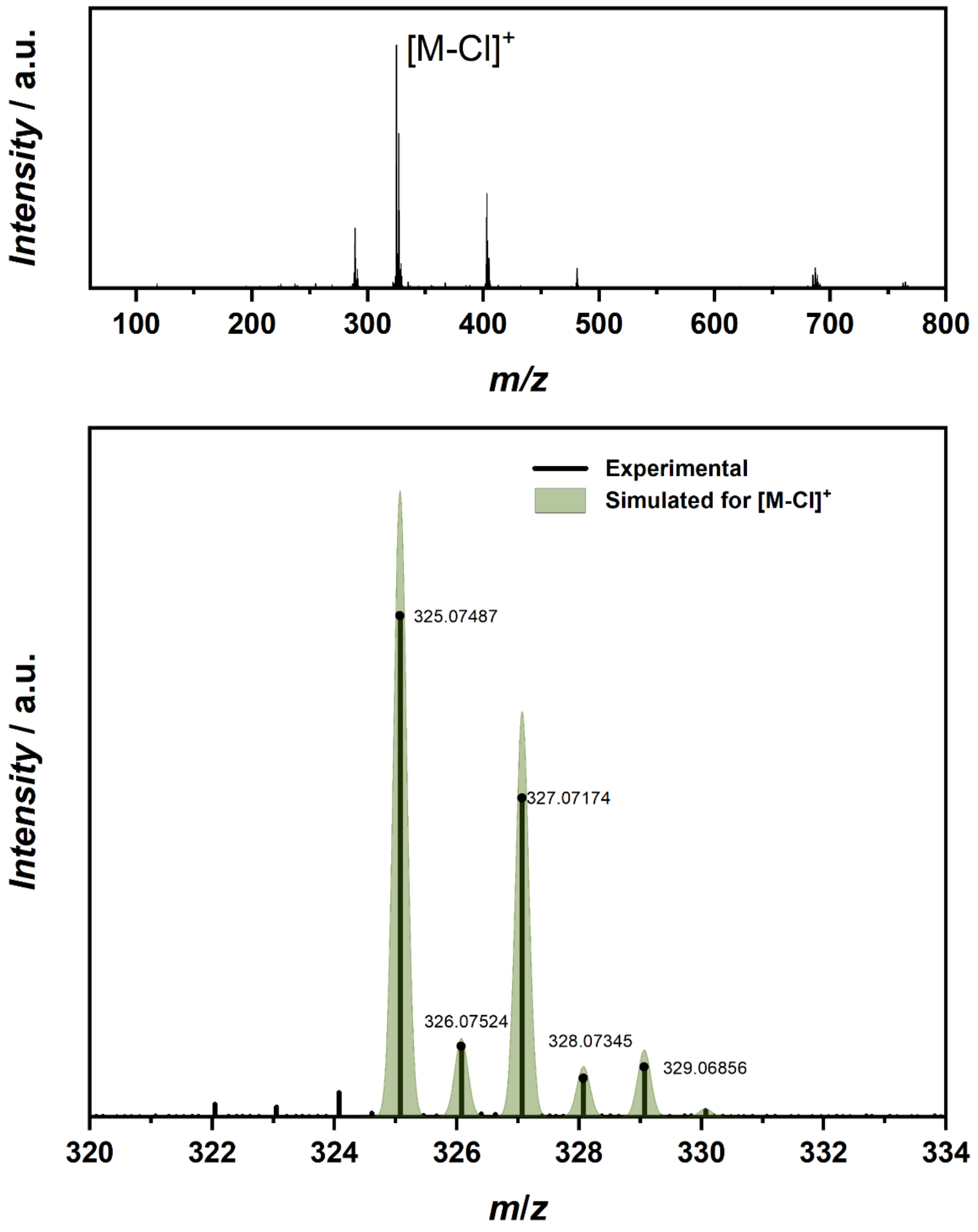

Figure S13 – ESI positive mass spectrum of **1** (top) with enlargement of the region of the molecular ion [M-Cl]$^+$ (bottom). Colour code: Experimental (black) and calculated (green).

*[Mn^III^(cyclam)(OH$_2$)(OTf)][OTf]$_2$ (**2**)*

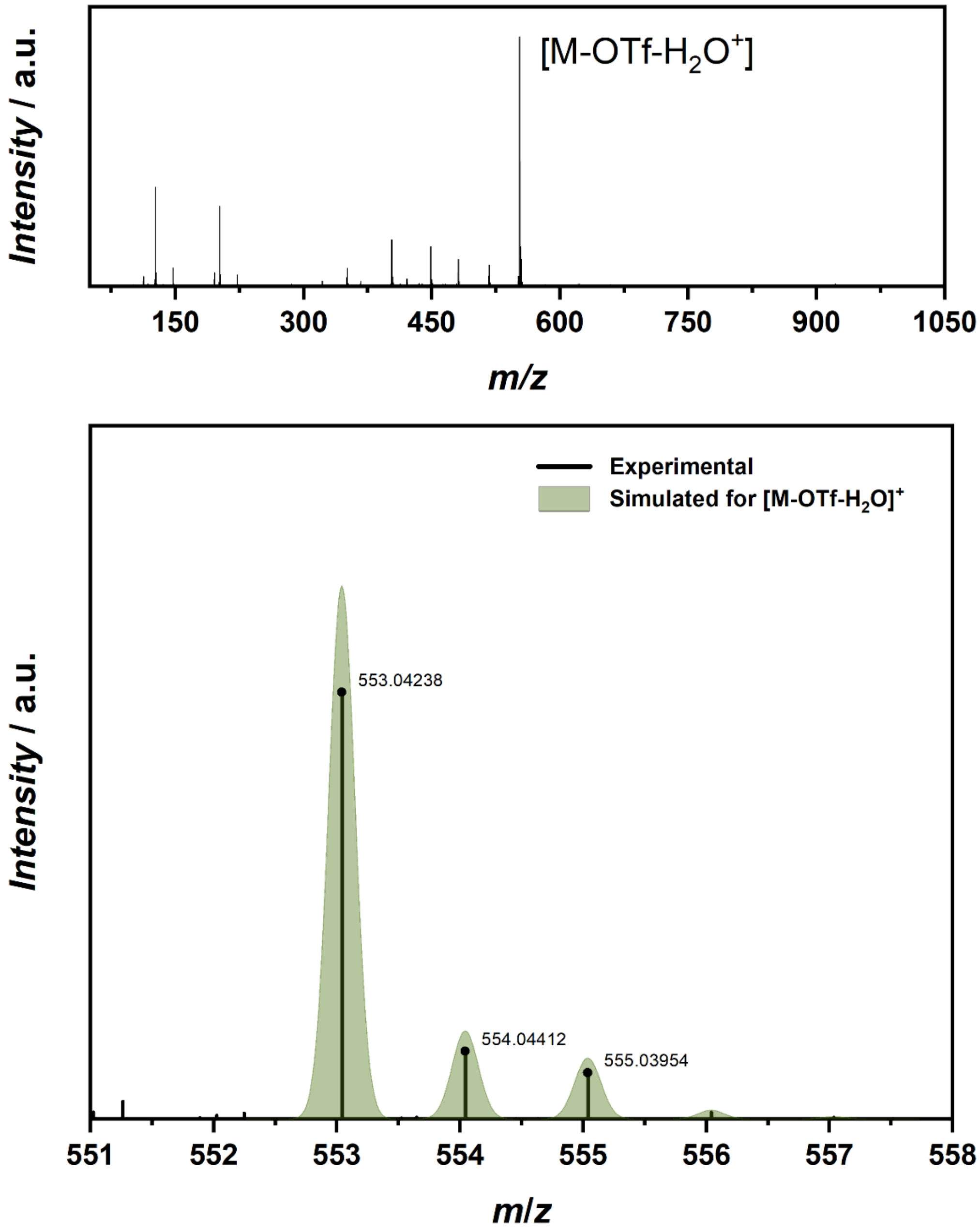

Figure S14 – ESI positive mass spectrum of **2** (top) with enlargement of the region of the molecular ion [M-OTf-H$_2$O]$^+$ (bottom). Colour code: Experimental (black) and calculated (green).

Infrared Spectroscopy

*[Mn^{III}(cyclam)Cl_2][Cl] (**1**)*

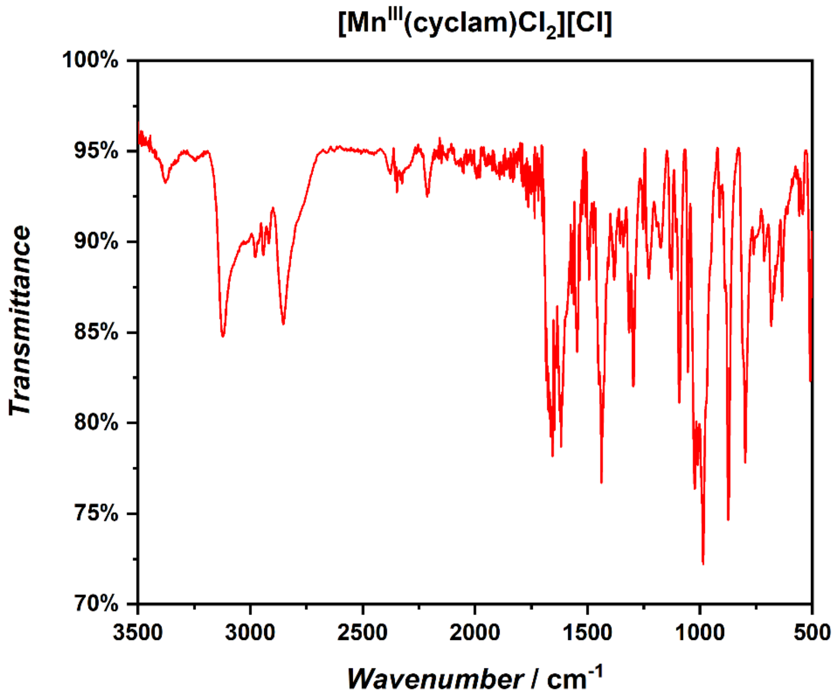

Figure S15 – Infrared spectrum of **1** between 3500-500 cm^{-1}.

*[Mn^{III}(cyclam)(OH_2)(OTf)][OTf]$_2$ (**2**)*

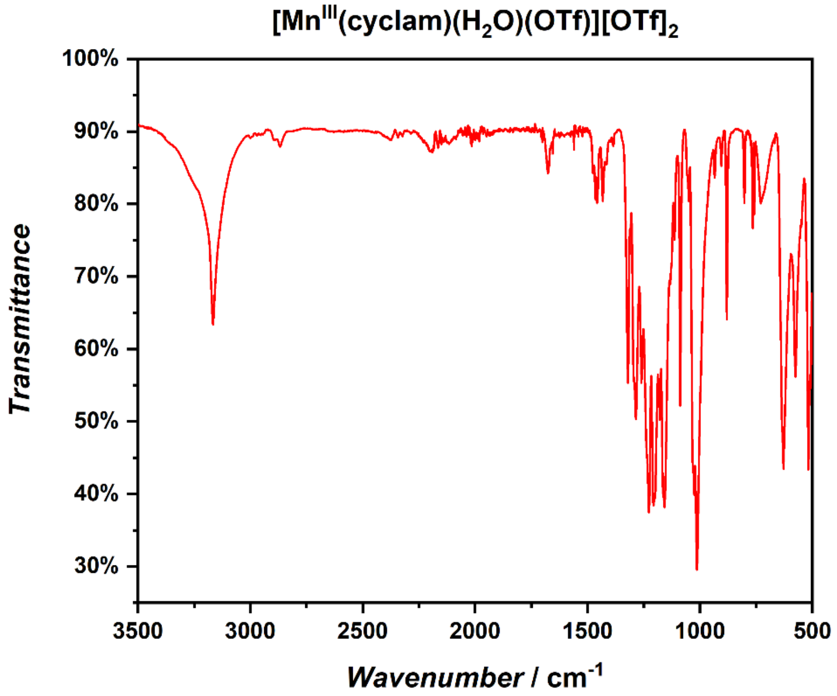

Figure S16 – Infrared spectrum of **2** between 3500-500 cm^{-1}.

Single Crystal X-Ray Crystallography

[Mn^{III}(cyclam)(OH$_2$)(OTf)][OTf]$_2$ (2)

A suitable crystal of **2** with dimensions 0.66 × 0.41 × 0.22 mm^3 was selected and mounted on a Bruker SMART APEXII diffractometer. The crystal was kept at a steady T = 120.15 K during data collection. The structure was solved with the ShelXT solution program using iterative methods and by using Olex2 as the graphical interface. The model was refined with ShelXL using full matrix least squares minimisation on F^2. Details are provided in Table S2-S4.[2-4]

Table S3 – Crystallographic details for compound **2**.

Empirical formula	$C_{26}H_{52}F_{18}Mn_2N_8O_{20}S_6$
Formula weight	1440.99
Crystal system	*Triclinic*
Space group	$P\bar{1}$
a / Å	8.8973(2)
b / Å	11.6583(3)
c / Å	13.8947(4)
α / °	72.869(2)
β / °	85.934(2)
γ / °	75.887(2)
V / Å^3	1335.75(6)
Z, *Z′*	2, 1
D_c / g·cm^{-3}	1.791
μ(Mo$K\alpha$) / mm^{-1}	0.846
F(000)	732.0
Crystal size / mm^3	0.66 × 0.41 × 0.22
T / K	120.1(5)
Observed reflections	9942 (9322)
R_{int}	0.0317
Parameters	367
GOF	1.051
R_1[a,b]	0.0301 (0.0378)
wR_2[c]	0.0696 (0.0733)
CCDC Number	2213596

a $R_1 = \sum(|F_0| - |F_c|)/\sum|F_0|$

b Values in parentheses for reflections with I > 2s(I).

c $wR_2 = \left[\sum w(F_0^2 - F_c^2)^2/\sum w(F_0^2)^2\right]^{1/2}$

The reaction of $Mn(OTf)_2$ with cyclam under a $HOTf_{(aq)}$ solution, following by slow evaporation of the mother liquor at room temperature, produces crystals of $[Mn^{III}(cyclam)(OTf)(OH_2)][OTf]_2$ (**2**) in approximately 16% yield. Complex **2** crystallises in the triclinic space group $P\bar{1}$ (Table S1) with two half-occupied, independent Mn^{III} centres in the asymmetric unit (Figure S17). Each centre is six-coordinated with the cyclam macrocycle in the equatorial plane and either two triflate anions or two water molecules in the axial positions. The first Mn^{III} in the asymmetric unit contains half of the macrocycle coordinating the Mn ion on the equatorial positions along with a triflate anion on the axial position; the second Mn ion also contains half of the macrocycle, but the axial position is occupied by a water molecule. The rest of the structure contains two triflate anions to balance the charge. Pertinent bond lengths and angles are provided in Tables S3-4.

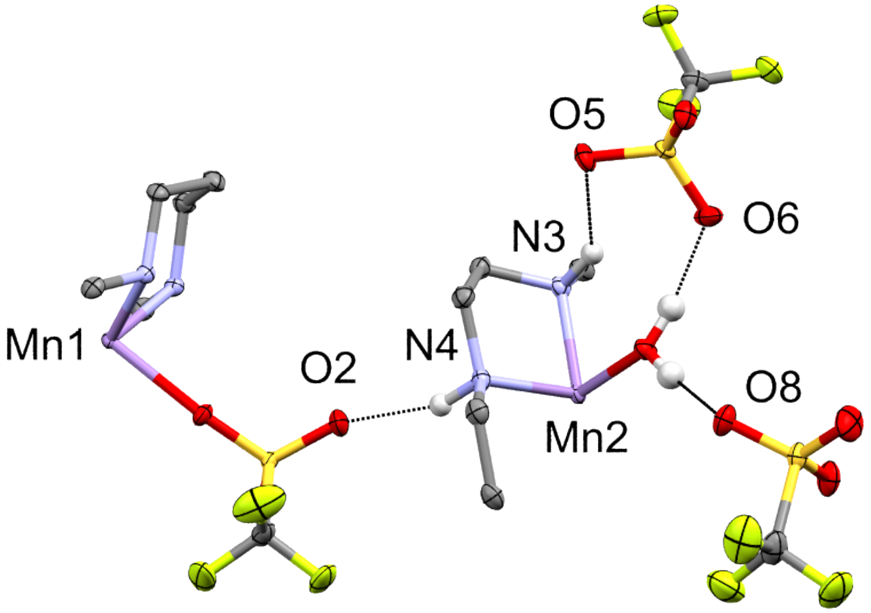

Figure S17 – Asymmetric unit of complex **2** with black lines indicating the H-bonds within the structure. Hydrogen atoms have been removed for simplicity (except those involved in H-bonding). Colour code: C, grey; N, light blue; O, red; Mn, lilac; S, yellow; F, light green.

All three triflate anions H-bond with neighbouring complex molecules. Two O-atoms from triflate anions (O2 and O5) are H-bonded with the NH groups from the macrocycle (N4 and N3, respectively). Two other O-atoms (O6 and O8) are H-bonded with the water molecule coordinated to the Mn^{III} centre (Figure S17). In the extended structure, the complex molecules pack in layers of either the triflate-coordinated or the aquo-coordinated molecules.

Table S4 – Selected bond distances (Å) for **2**.

Atom	Atom	Length / Å	Atom	Atom	Length / Å
Mn1	O1[a]	2.1753(9)	F6	C12	1.3270(17)
Mn1	O1	2.1753(9)	F3	C11	1.3239(15)
Mn1	N2[a]	2.0300(10)	F5	C12	1.3281(16)
Mn1	N2	2.0300(10)	F4	C12	1.3364(17)
Mn1	N1	2.0346(10)	F1	C11	1.3263(18)
Mn1	N1[a]	2.0346(10)	F9	C13	1.3333(18)
Mn2	O4[b]	2.2050(9)	F8	C13	1.332(2)
Mn2	O4	2.2050(9)	F7	C13	1.3242(19)
Mn2	N4	2.0247(10)	F2	C11	1.321(2)
Mn2	N4[b]	2.0247(10)	N4	C8	1.4960(17)
Mn2	N3	2.0408(11)	N4	C9	1.4858(17)
Mn2	N3[b]	2.0408(11)	N2	C5	1.4959(16)
S1	O1	1.4679(9)	N2	C4	1.4899(16)
S1	O2	1.4311(10)	N3	C6	1.4917(16)
S1	O3	1.4262(10)	N3	C7	1.4952(16)
S1	C11	1.8247(14)	N1	C1	1.4953(16)
S2	O7	1.4376(10)	N1	C2	1.4887(16)
S2	O5	1.4427(10)	C5	C1[a]	1.5108(18)
S2	O6	1.4515(10)	C8	C7	1.5148(19)
S2	C12	1.8198(14)	C4	C3	1.5230(18)
S3	O8	1.4490(10)	C6	C10[b]	1.5246(19)
S3	O10	1.4462(11)	C2	C3	1.5190(19)
S3	O9	1.4328(12)	C9	C10	1.5224(19)
S3	C13	1.8244(17)			

Table S4 – Selected angles (°) for **2**.

Atom	Atom	Atom	Angle / °	Atom	Atom	Atom	Angle / °
O1[a]	Mn1	O1	180	O9	S3	O8	115.32(7)
N2[a]	Mn1	O1[a]	86.40(4)	O9	S3	O10	116.11(7)
N2	Mn1	O1	86.40(4)	O9	S3	C13	103.98(8)
N2	Mn1	O1[a]	93.60(4)	S1	O1	Mn1	153.58(6)
N2[a]	Mn1	O1	93.60(4)	C8	N4	Mn2	107.02(7)
N2	Mn1	N2[a]	180	C9	N4	Mn2	117.73(8)
N2	Mn1	N1	94.06(4)	C9	N4	C8	112.75(10)
N2[a]	Mn1	N1	85.94(4)	C5	N2	Mn1	106.80(7)
N2[a]	Mn1	N1[a]	94.06(4)	C4	N2	Mn1	118.07(8)
N2	Mn1	N1[a]	85.94(4)	C4	N2	C5	113.17(9)
N1	Mn1	O1	91.31(4)	C6	N3	Mn2	117.99(8)
N1[a]	Mn1	O1[a]	91.31(4)	C6	N3	C7	113.05(10)
N1	Mn1	O1[a]	88.69(4)	C7	N3	Mn2	106.39(8)
N1[a]	Mn1	O1	88.69(4)	C1	N1	Mn1	106.11(7)
N1	Mn1	N1[a]	180	C2	N1	Mn1	117.89(7)
O4[b]	Mn2	O4	180	C2	N1	C1	112.91(10)

[a] $-X, 2-Y, Z$
[b] $1-X, 1-Y, 1-Z$

Table S4 (continuation)

Atom	Atom	Atom	Angle / °	Atom	Atom	Atom	Angle / °
N4[b]	Mn2	O4	88.59(4)	N2	C5	C1[a]	107.61(9)
N4[b]	Mn2	O4[b]	91.41(4)	N4	C8	C7	107.91(10)
N4	Mn2	O4[b]	88.59(4)	N2	C4	C3	111.41(10)
N4	Mn2	O4	91.41(4)	N3	C6	C10[b]	112.38(11)
N4[b]	Mn2	N4	180	N1	C1	C5[a]	107.79(10)
N4	Mn2	N3	85.90(4)	N1	C2	C3	112.44(10)
N4[b]	Mn2	N3[b]	85.90(4)	N3	C7	C8	107.66(10)
N4[b]	Mn2	N3	94.10(4)	N4	C9	C10	111.73(11)
N4	Mn2	N3[b]	94.10(4)	C2	C3	C4	114.82(10)
N3[b]	Mn2	O4	89.81(4)	C9	C10	C6[b]	114.59(11)
N3	Mn2	O4[b]	89.81(4)	F6	C12	S2	110.96(10)
N3	Mn2	O4	90.19(4)	F6	C12	F5	108.77(11)
N3[b]	Mn2	O4[b]	90.19(4)	F6	C12	F4	107.87(12)
N3[b]	Mn2	N3	180.00(5)	F5	C12	S2	110.54(10)
O1	S1	C11	101.26(6)	F5	C12	F4	108.15(12)
O2	S1	O1	113.13(6)	F4	C12	S2	110.46(9)
O2	S1	C11	103.80(7)	F3	C11	S1	110.74(10)
O3	S1	O1	113.42(6)	F3	C11	F1	107.70(12)
O3	S1	O2	117.30(7)	F1	C11	S1	111.13(11)
O3	S1	C11	105.70(7)	F2	C11	S1	110.62(10)
O7	S2	O5	115.38(6)	F2	C11	F3	108.60(13)
O7	S2	O6	115.05(6)	F2	C11	F1	107.95(14)
O7	S2	C12	103.85(6)	F9	C13	S3	110.84(11)
O5	S2	O6	113.63(6)	F8	C13	S3	111.33(11)
O5	S2	C12	103.93(6)	F8	C13	F9	107.68(15)
O6	S2	C12	102.86(6)	F7	C13	S3	111.57(13)
O8	S3	C13	102.70(7)	F7	C13	F9	107.56(13)
O10	S3	O8	113.79(6)	F7	C13	F8	107.68(14)
O10	S3	C13	102.37(8)				

[a] $-X, 2-Y, Z$
[b] $1-X, 1-Y, 1-Z$

Powder X-Ray Crystallography

*[Mn^{III}(cyclam)Cl_2][Cl] (**1**)*

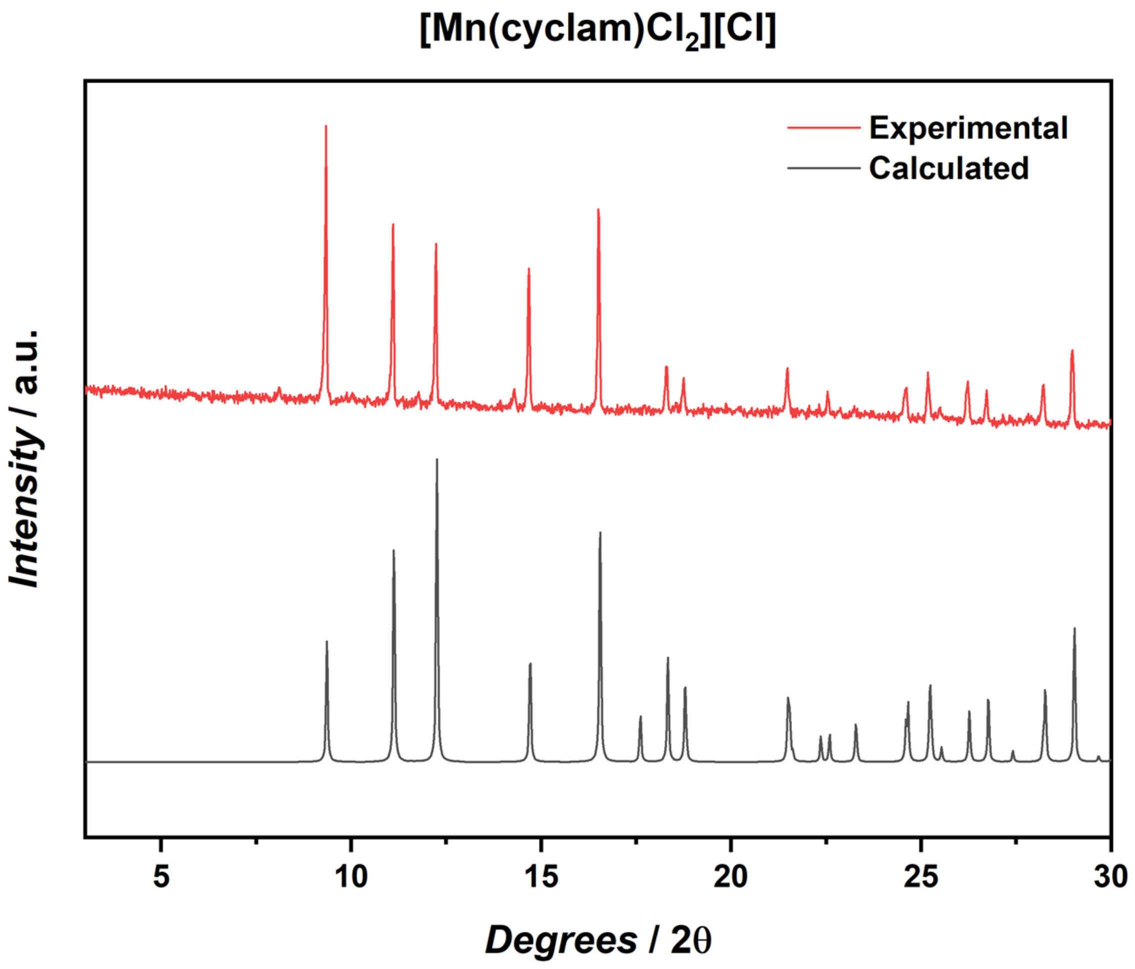

Figure S18 – Powder X-ray diffraction of **1**. Experimental data (red) obtained at room temperature and calculated (black) data from crystal structure (CCDC 1166174).[5]

*[Mn^{III}(cyclam)(OH_2)(OTf)][OTf]$_2$ (**2**)*

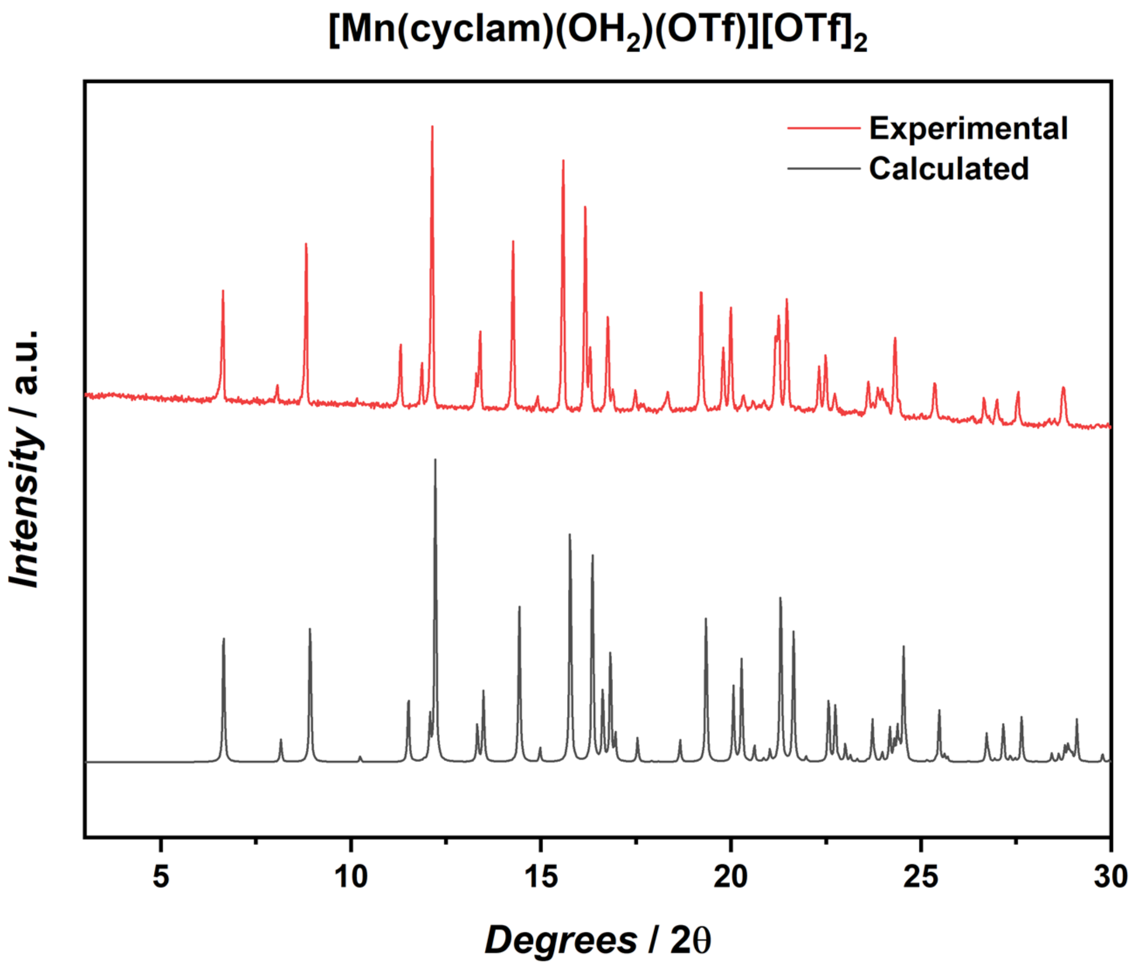

Figure S19 – Powder X-ray diffraction of **2**. Experimental data (red) obtained at room temperature and calculated (black) data from crystal structure (CCDC 2213596).

References

1. CrysAlisPRO Oxford Diffraction /Agilent Technologies UK Ltd, 2015.
2. OLEX2: a complete structure solution, refinement and analysis program. O. V. Dolomanov, L. J. Bourhis, R. J. Gildea, J. A. K. Howard and H. Puschmann, *J. Appl. Crystallogr.*, 2009, **42**, 339. DOI : 10.1107/S0021889808042726
3. SHELXT - Integrated space-group and crystal-structure determination. G. M. Sheldrick, *Acta Crystallogr. Sect. A Found. Adv.*, 2015, **71**, 3. DOI: 10.1107/S2053273314026370
4. Crystal structure refinement with SHELXL. G. M. Sheldrick, *Acta Crystallogr. Sect. C Struct. Chem.*, 2015, **71**, 3. DOI: 10.1107/S2053229614024218
5. Dichloro(1,4,8,11-tetraazacyclotetradecane)manganese(III) chloride: *cis–trans* isomerisation evidenced by infrared and electrochemical studies. F. Létumier, G. Broeker, J.-M. Barbe, R. Guilard, D. Lucas, V. Dahaoui-Gindrey, C. Lecomte, L. Thouin and C. Amatore, *J. Chem. Soc., Dalton Trans*. 1998, 2233. DOI: 10.1039/A800824H
6. Manganese (III) cyclam complexes with aqua, iodo, nitrito, perchlorato and acetic acid/acetato axial ligands. S. Mossin, H. O. Sørensen, H. Weihe, J. Glerup and I. Søtofte*, Inorg. Chim. Acta*, 2005, **358**, 4, 1096. DOI: 10.1016/j.ica.2004.10.005
